# SOCS5, targeted by miR-155-5p, plays a negative regulatory role in pulmonary hypertension through inhibiting JAK2/STAT3 signaling pathway

**DOI:** 10.1186/s12890-024-02857-6

**Published:** 2024-01-24

**Authors:** Lili Sun, Lihua Liu, Dongxue Liang, Linlin Liu

**Affiliations:** Ward of Respiratory and Critical Care Department, The First Affiliated Hospital of Jinzhou Medical University, No. 2 Section 5 Renmin Street, Jinzhou, Liaoning P.R. China

**Keywords:** Pulmonary hypertension, SOCS5, miR-155-5p

## Abstract

Pulmonary hypertension (PH) is a chronic pulmonary vascular disease and causes massive deaths. Here, we intended to investigate the function and mechanism of SOCS5 in PH. We engineered a hypoxia-induced PH model in mice. HE staining were implemented to evaluate pathological alterations in the lung tissues. The potential mechanism of SOCS5 in regulating hypoxia-induced pulmonary artery smooth muscle cell (PASMC) function was explored in vitro. RT-qPCR and western blot revealed that the level of SOCS5 was decreased both in PH mice and hypoxia-induced HPASMCs. Functional assays were performed for confirming the role of SOCS5 in modulating the cell phenotype and JAK2/STAT3 pathway in HPASMCs. Results revealed that overexpression of SOCS5 suppressed proliferation, migration and contraction of HPASMCs and negatively regulated the JAK2/STAT3 signaling pathway in HPASMCs under hypoxia in vitro, while knockdown of SOCS5 accelerated it. As evidenced by mechanism studies, SOCS5 was targeted and regulated by miR-155-5p, hence affecting on HPASMC proliferation, migration and contraction. These outcomes indicated that the decreased level of SOCS5 in hypoxia-induced HPASMCs promoted the cell proliferation, cell migration, and cell contraction through activating JAK2/STAT3 signaling pathway. Moreover, SOCS5 was targeted by miR-155-5p. All in all, our work hinted that miR-155-5p/SOCS5/JAK2/STAT3 axis played a crucial part in PH.

**Supplementary Information:**

The online version contains supplementary material available at 10.1186/s12890-024-02857-6.

## Introduction

Pulmonary hypertension (PH) is a chronic pulmonary circulatory system disease caused by multiple factors. It is characterized by elevated pulmonary pressure, changes in pulmonary circulation structure, and the formation of vascular occlusions [1]. Long-term persistent PH will eventually lead to right ventricular dysfunction and even death due to heart failure [2]. With the emergence of targeted therapies, greatly improving the prognosis of patients with PH, however, PH remains a progressive disease with significant morbidity and mortality [3]. The current treatment of PH is mainly the use of pulmonary vasodilators, which improve symptoms and reduce hospital admissions [4]. However, pulmonary vasodilators don't aim at the key features of PH pathogenesis. Therefore, in-depth study of its pathogenesis will be helpful to explore new therapeutic methods.

The pathogenesis of PH mainly includes persistent pulmonary vasoconstriction, pulmonary vascular remodeling and microthrombus formation [5]. In hypoxic PH, abnormal proliferation and migration of pulmonary arterial smooth muscle cells (PASMCs) are the main pathological changes leading to pulmonary vascular structural remodeling [6]. Studies have shown that IL-19 inhibited the proliferation of vascular smooth muscle cells (VSMCs) and is accompanied by increased SOCS5 expression [7]. Nevertheless, whether SOCS5 expression affects phenotypes of PASMCs in PH is unknown.

SOCS5 is one of the members of the SOCS family, located on chromosome 2p21 and widely expressed in multiple tissues and cells. It plays a regulatory role in disease progression by affecting multiple signaling pathways. Studies have shown that SOCS5 was able to inhibit the JAK2/STAT3 pathway [8–11]. SOCS5 improved flu-induced airway infection by inhibiting EGFR signaling [12]. Up-regulation of SOCS5 was associated with alleviation of airway inflammation and remodeling in asthmatic mice [13]. Moreover, targeted inhibition of SOCS5 by upregulated miR-132 promoted the progression of chronic obstructive pulmonary disease [14]. However, little is known about how SOCS5 regulates signal transduction in PH.

JAK2, a member of the JAKs family, is activated by catalyzing tyrosine phosphorylation of STATs, thereby mediating cell proliferation, differentiation, apoptosis and immune regulation [15]. JAK2/STAT3 signaling is negatively regulated by SOCS family [11]. Most important of all, inhibition of JAK2/STAT3 pathway activation has been reported to be able to delay the development of PH [16–19]. Through analyzing a dataset (GSE15197) from the Gene Expression Omnibus (GEO) database (https://www.ncbi.nlm.nih.gov/geo), we found that SOCS5 expression was reduced in lung tissue from PH patients. Therefore, we assumed that reduction of SOCS5 may relieve the inhibition of JAK2/STAT3 pathway, in turn, promote the development of PH.

Besides, increasing evidence has shown that microRNAs (miRNAs) are also important for the occurrence and development of diseases. Through database prediction, we found that SOCS5 may be a target gene of miR-155. It has been reported that miR-155 was associated with hypertension, angiogenesis and atherosclerosis [20, 21]. The expression of miR-155 is up-regulated in pulmonary hypertension [22].

In the current study, we explored the mechanism of SOCS5/JAK2/STAT3 axis in PH and further verified whether SOCS5 was regulated by miR-155. Our study is of great significance for improving the therapeutic effect of PH patients.

## Material and methods

### Animal models and hemodynamic analysis

Male C57BL/6 mice (8-week-old) were purchased from Liaoning Changsheng Biotechnology. After a week of acclimatization, mice were randomly divided into two groups (six mice/group): the control mice were exposed to normal air (normoxia), while the PH mice were exposed to mix air containing 10% oxygen and 90% nitrogen (hypoxia) for four weeks as previously described [23]. Briefly, the mice were housed in a chamber with a mixture of oxygen and nitrogen and the oxygen concentration was monitored and controlled. This study was conducted in accordance with the Guideline for the Care and Use of Laboratory Animals from National Institutes of Health (NIH) and ARRIVE. All experiments were undertaken with approval of the Animal Ethics Committee of Jinzhou Medical University.

After four weeks, the mice were anaesthetized by isoflurane. The right ventricular systolic pressure (RVSP) of mice was measured by closed-chest insertion into the right ventricle via an external jugular vein approach of a PE catheter (Smiths Medical, USA) connected to a pressure transducer in the spontaneously breathing, anesthetized animal [24]. Following euthanasia, the murine lungs and hearts were collected, then right ventricular (RV) and left ventricular including the interventricular septa (LV + S) were weighed. The right ventricular hypertrophy index (RVHI) was expressed as RV/(LV + S).

### Cell culture

Human pulmonary arterial smooth muscle cells (HPASMCs) were purchased from iCell Bioscience Inc, Shanghai (China) and cultured in ICell primary smooth muscle cell low serum culture system (iCell Bioscience Inc, Shanghai, China) at 3 °C and 5% CO_2_. For transfection, when the confluence of cells reached 70%, HPASMCs were then transfected with an overexpression plasmid or small interfering RNA (siRNA) targeting SOCS5 using liposome 8000 (Beyotime Institute of Biotechnology, China) at 37 °C for 24 h. For hypoxic treatment, HPASMCs were cultured under hypoxia conditions (3% oxygen, 5% carbon dioxide, 92% nitrogen) for indicated time period after 3 h of serum starvation.

### Haematoxylin and eosin (H&E) staining

Lung tissues from six animals per group were respectively subjected to H&E staining. After fixed in 4% paraformaldehyde, the lung tissues were embedded in paraffin and cut into 5 μm sections. Haematoxylin (Beijing Solarbio Science & Technology Co., Ltd, China) and eosin (Sangon biotech, China) staining was used to visualize lung tissue lesions. For the quantification of vascular medial wall thickness, two sections of lung tissue from each animal were used for visualization, then two fields were taken from each section. We measured a 50–150 μm long artery in one field of view. A microscope (BX53, Olympus Corporation, Japan) was used for observation and photographs were taken using a camera system (DP73, Olympus Corporation, Japan).

### Immunohistochemistry

For immunohistochemistry, the paraffin slices were subjected to dewaxing and hydration firstly. After that, the slides were put into antigen retrieval solution and heated for 10 min to retrieve the antigen. The slides were blocked with 1% BSA (Sangon biotech, China) for 15 min at room temperature after treatment with 3% hydrogen peroxide (Sinopharm, China) for 15 min. Then the p-JAK2 (AP0531, 1:50, ABclonal Technology Co., Ltd., China) and p-STAT3 (AP0705, 1:50, ABclonal Technology Co., Ltd., China) primary antibodies were added to the slides for incubation overnight at 4 °C. The HRP labeled goat anti-rabbit IgG (D110058, 1:200, Sangon biotech, China) secondary antibody was used for incubation for 60 min at room temperature. DAB chromogenic solution (Beijing Solarbio Science & Technology Co.,Ltd, China) was used to develop the color, and the sections were counterstained with hematoxylin (Beijing Solarbio Science & Technology Co.,Ltd, China). Finally, the slices were observed under the microscope (BX53, Olympus Corporation, Japan) and photographed.

### Quantitative reverse transcription PCR (RT-qPCR)

Isolation of total RNA from mouse lung tissues or cells was performed using TRIpure lysate (Bioteke Corporation, China). RNA concentration was determined using ultraviolet spectrophotometer (NANO 2000, Thermo Fisher Scientific, USA). Total RNA was reverse-transcribed to cDNA using random primer and BeyoRT II M-MLV reverse transcriptase (Beyotime Institute of Biotechnology, China) for mRNA analysis and miRNA First strand cDNA Synthesis (tail-adding) kit (Sangon biotech, China) for miRNA analysis. qPCR was performed with cDNA using 2 × Taq PCR MasterMix (Beijing Solarbio Science & Technology Co.,Ltd, China), DNA binding SYBR Green dye (Beijing Solarbio Science & Technology Co.,Ltd, China) and specific primers for each target genes. The sequences of the forward and reverse primer were listed in Table 1. All PCR reaction data were obtained from the fluorescence quantitative instrument (Exicycler™ 96, BIONEER Corporation, Korea). The cycling conditions were as follows: initial denaturation at 94 °C for 5 min, followed by 40 cycles of denaturation at 94 °C for 10 s, annealing at 60 °C for 15 s, and extension at 72 °C for 25 s. The β-actin gene was used as the reference and the U6 gene was used as the internal control for miR-155p-5p.

**Table 1:** Information of the primers for RT-qPCR

Primer name	Sequence	Product length	Gene ID
mus SOCS1 F	CGCTCCCACTCCGATTACC	284	NM_009896.2
mus SOCS1 R	TGCTCCAGCAGCTCGAAAA		
mus SOCS2 F	CTAACCTGCGGATTGAG	211	NM_007706.4
mus SOCS2 R	AGAGTGGGTGCTGATGT		
mus SOCS3 F	GCGGATTCTACTGGAGCG	156	NM_007707.3
mus SOCS3 R	GGATGCGTAGGTTCTTGGTC		
mus SOCS5 F	CAGGACACGGTGGGTTT	112	NM_019654.2
mus SOCS5 R	GGCATTTCTCCAGCATTA		
homo SOCS5 F	CTCCTGGAATGACTGAA	150	NM_014011.5
homo SOCS5 R	GTCTGCTAACATGGGTAT		
hsa-miR-155-5p F	TTAATGCTAATCGTGATAGGGGTT		MIMAT0000646

### Western blot

Total proteins were extracted from homogenized mouse lung tissues or HPASMCs and the protein concentration was measured using a BCA protein concentration assay kit (Beyotime Institute of Biotechnology, China). The protein samples were separated by SDS‐PAGE and transferred to PVDF membranes, followed by blocked with 5% skim milk powder solution (Inner Mongolia Yili Industrial Group Co., Ltd., China)/3% BSA (Labgic Technology Co., Ltd., China) for 1 h. The molecular weights of protein blots can be distinguished by the loading protein marker. The PVDF membranes were cut according to the protein marker prior to hybridization with primary antibodies. Then membranes were incubated overnight at 4 °C with primary antibodies against SOCS5 (A7952, 1:1,000), JAK2 (A11497, 1:1,000), p-JAK2 (AP0531, 1:1,000), STAT3 (A1192, 1:1,000), p-STAT3 (AP0705, 1:1,000, all from ABclonal Technology Co., Ltd., China), β-actin (sc-47778, 1:1,000, santa cruz biotechnology, inc., USA) and incubated with HRP (1:5,000, Beyotime Institute of Biotechnology, China) bound goat anti-rabbit IgG (A0208)/goat anti-mouse IgG (A0216) at 37 °C for 45 min. All antibodies were diluted with 5% skim milk powder solution. Finally, the protein bands were detected by the ECL system (Beyotime Institute of Biotechnology, China).

### Immunofluorescence

For immunofluorescence double staining, the slides with 5 μm paraffin sections were deparaffinized in xylene, rehydrated, incubated in antigen retrieval solution for 10 min and blocked with 1% BSA (Sangon biotech, China) for 15 min. The slides were incubated with primary antibodies against SOCS5 (A7952, 1:50, ABclonal Technology Co., Ltd., China), α‐SMA (67735–1-Ig, 1:200, Proteintech Group, Inc., China), CD31 (sc-376764, 1:50, Santa Cruz Biotechnology, USA) and Vimentin (BF8006, 1:100, Affinity Biosciences LTD, China) at 4 °C overnight. Then, the slides were incubated with secondary antibodies Cy3-labeled goat anti-rabbit IgG (#4413, 1:500/1:200, Cell Signaling Technology, USA) and fluorescein isothiocyanate (FITC)-labeled goat anti-mouse IgG (#4408, 1:500/1:200, Cell Signaling Technology, USA) at room temperature for 90 min.

For immunofluorescence single staining, the slides were fixed in 4% paraformaldehyde (China National Medicines Corporation Ltd.) for 15 min, incubated with 0.1% Triton X‐100 (Beyotime Institute of Biotechnology, China) at room temperature for 30 min and then blocked with 1% BSA (Sangon biotech, China) for 15 min. The slides were incubated with primary antibody against α‐SMA (67,735–1-Ig, 1:200, Proteintech Group, Inc., China) at 4 °C overnight. Then, the slides were incubated with secondary antibody fluorescein isothiocyanate (FITC)-labeled goat anti-mouse IgG (#4408, 1:500, Cell Signaling Technology, USA) at room temperature for 60 min.

At the end, both for single and double staining, DAPI was used for nuclear staining. A fluorescence microscope (BX53, Olympus Corporation, Japan) was used for observation and photographs were taken using a camera system (DP73, Olympus Corporation, Japan).

### 3-(4, 5-Dimethylthiazol-2-yl)-2, 5-diphenyltetrazolium Bromide (MTT) assay

HPASMCs (4 × 10^3^ cells/well) were seeded into 96‐well plates. Adding 50 μl MTT dye (Jiangsu KeyGEN BioTECH Corp., Ltd, China) into each well to incubate for 4 h at 37 °C after a specific time or treatment. Subsequently, 150 μl dimethyl sulfoxide (DMSO, Jiangsu KeyGEN BioTECH Corp., Ltd, China) was added and cell viability (OD490) was detected using a microplate reader (800TS, Bioteke Corporation, China).

### 5‐ethynyl‐2’‐deoxyuridine (EdU) incorporation assay

HPASMCs were transfected for 24 h, starved for 3 h, treated with normoxia or hypoxia for 72 h, EdU incorporation assay was performed using kFluor647 Click-iT EdU imaging detection kit (Jiangsu KeyGEN BioTECH Corp., Ltd, China) according to the manufacturer's instructions. Hoechst 33,342 was used for nucleus counterstaining. The EdU‐positive cells were visualized under a fluorescent microscope (IX53, Olympus Corporation, Japan) and quantified.

### Transwell migration assay

The migration of HPASMCs was measured by transwell assay. In brief, HPASMCs were resuspended in serum‐free medium and then cell suspension (200 μl) was seeded into the upper chamber. Medium containing 10% FBS (800 μl) was added in the lower chamber. Following incubation for 24 h at 37 °C in an incubator with 5% CO_2_, cells in the lower chamber were fixed with 4% paraformaldehyde (Shanghai Aladdin Bio-Chem Technology Co., Ltd, China) for 20 min at room temperature and stained with 0.5% crystal violet (Amresco, USA) for 5 min. An inverted microscope (IX53, Olympus Corporation, Japan) (200 ×) was used to count the stained cells in three randomly selected fields.

### Cell contraction assay

HPASMCs were starved for 3 h, mixed with collagenase I (Labgic Technology Co., Ltd., China) and 2 × DMEM medium (Wuhan Servicebio Technology Co., Ltd, China), placed into a 24-well plate, followed by incubation at 37 °C until the gel was polymerized. Finally, the collagen gel size changes relative to the initial collagen gel size were imaged and quantified.

### Measurement of Ca^2+^

Fluo-4 AM (Shanghai Maokang Biotechnology Co., Ltd, China), a fluorescent dye that can specifically bind to Ca^2+^, was assessed to detect Ca^2+^ in cells. HPASMCs were cultured with 2 μM Fluo-4 AM in 24-well plates and incubated at 37 °C for 30 min. A fluorescence microscope (IX53, Olympus Corporation, Japan) was used to observe the target signals and random fields were imaged.

### Luciferase reporter assay

TargetScan (http://www.targetscan.org/vert_72/) and miRDB (www.mirdb.org/) databases were employed to predict the target sites of hsa-miR-155-5p in the SOCS5 3′ untranslated region (3′-UTR). The SOCS5 3′-UTR containing miR-155-5p binding sites was subcloned into the pmirGLO luciferase reporter vector (General Biosystems (Anhui) Co., Ltd, China) to construct the wild type (wt) luciferase reporter vector (SOCS5 wt) and the mutant (mut) sequence of SOCS5 was subcloned into the luciferase reporter vector to construct the mutant luciferase reporter vector (SOCS5 mut). For dual luciferase assay, the reporter plasmid and 45 pmol miR-155-5p mimic or NC mimic were co-transfected into the 293 T cells (Shanghai Zhong Qiao Xin Zhou Biotechnology Co., Ltd, China) with liposome 3000 (Invitrogen, USA), followed by incubation at 37 °C for 48 h. The luciferase activity was determined using a luciferase detection kit (Jiangsu KeyGEN BioTECH Corp., Ltd, China), according to the manufacturer’s instructions. The firefly luciferase activity was normalized to renilla luciferase activity.

### Statistical analysis

All data were statistically analyzed using GraphPad Prism 8.0.2 (GraphPad Software, Inc.) and presented as the mean ± SD (standard deviation). For two groups, differences were compared using an unpaired Student's t-test and for comparison among three groups, the data were analyzed using one-way analysis of variance (ANOVA). Statistical significance was defined as *P* < 0.05 or *P* < 0.01. All data were repeated at least three times.

## Results

### Reduced in lung tissue expression of SOCS5 in a mouse model of PH

**Fig. 1:**
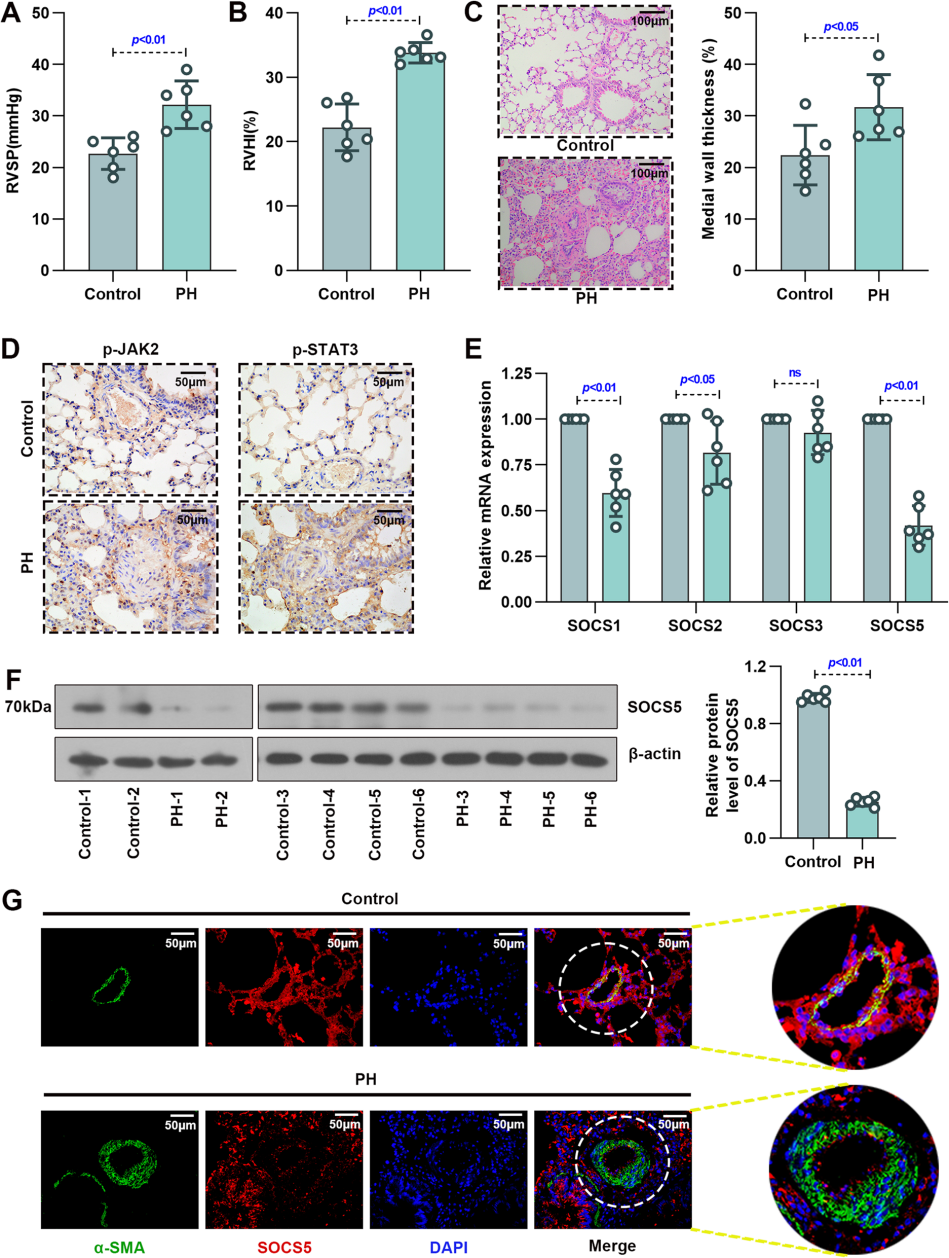
The hypoxia-induced PH mouse model was successfully established and the expression of p-JAK2 and p-STAT3 were increased, while the expression of SOCS5 was reduced in PH mice. **A**&**B** Increased RVSP and RVHI in lung sections from mice exposed to normoxia (Control) or hypoxia (PH) for 4 weeks (*n* = 6 each group). **C** Representative photographs were shown of H&E staining of pulmonary arteries from control and PH mice. Scale bar = 100 μm. The percentage of the inner wall thickness was quantified (*n* = 6 each group). **D** Representative images of IHC of pulmonary arteries from control and PH mice to detect p-JAK2 and p-STAT3. Scale bar = 50 μm. **E** SOCS1, SOCS2, SOCS3 and SOCS5 mRNA expression of whole lungs from control and PH mice (*n* = 6 each group). **F** Western blot analysis and quantification of SOCS5 in whole lungs of Control and PH mice (*n* = 6 each group). **G** Representative photographs of immunofluorescence staining for SOCS5 and α-SMA in whole lungs of control and PH mice. Scale bar = 50 μm. PH, pulmonary hypertension; RVSP, right ventricular systolic pressure; RVHI, right ventricular hypertrophy index; H&E, hematoxylin–eosin; IHC, immunohistochemistry

RVSP and RVHI were measured to determine the development of PH. The RVSP and RVHI were substantially elevated in mice with PH induced by exposure to hypoxia for 4 weeks compared with control mice exposed to normoxia (Fig. 1A&B). The mean percent medial wall thickness of pulmonary arteries significantly increased in PH group compared with corresponding normoxia control (Fig. 1C). These results indicated that the hypoxia-induced PH model was successfully established in mice.

In the mouse lung tissue, the expression of p-JAK2 and p-STAT3 were increased (Fig. 1D). Part of the SOCS family (SOCS1, SOCS2, SOCS5) mRNA expression significantly decreased on hypoxia exposure compared with normoxia controls; however, there was no difference in SOCS3 mRNA expression (Fig. 1E). Western blotting further confirmed that the SOCS5 protein expression was significantly decreased in the lung tissue of mice exposed to hypoxia compared with normoxia (Fig. 1F). Moreover, in both normoxic and hypoxic mice, immunofluorescence (IF) staining for SOCS5 protein was found in PASMCs, as shown by double-immunofluorescence staining for SOCS5 and α-SMA (a smooth muscle cell marker) of lung sections (Fig. 1G). SOCS5 protein was reduced in the PASMCs of hypoxic mice compared to the normoxic mice. As for immunofluorescence staining for SOCS5 and CD31, SOCS5 and Vimentin in whole lungs, the change of SOCS5 protein in PH group was not obvious compared with the control group (Fig. S2). These results suggested that SOCS5 expression was reduced in lung tissue especially in PASMCs of PH mice.

### Effects of hypoxia on SOCS5 expression in HPASMCs

**Fig. 2:**
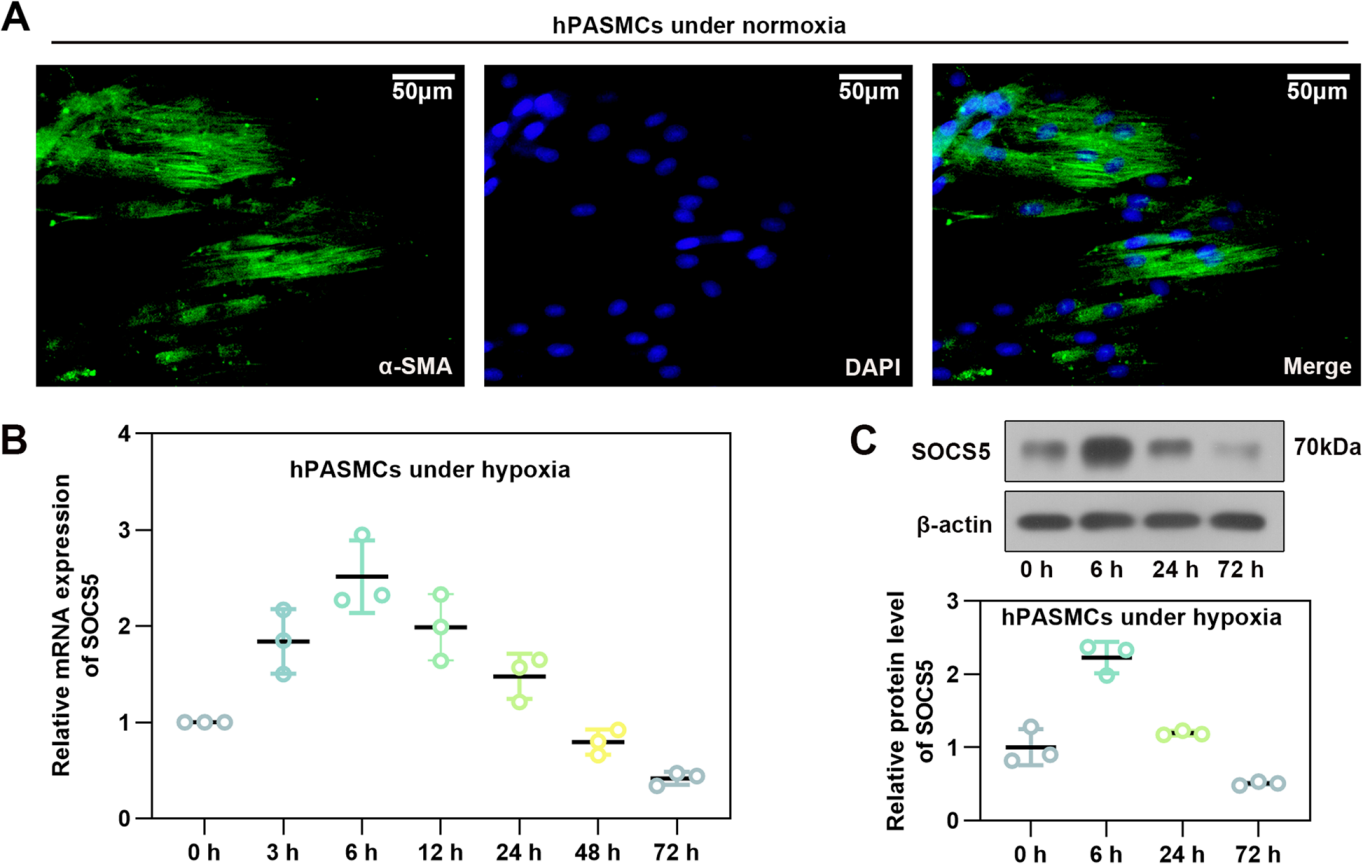
SOCS5 expression in hypoxia-induced HPASMCs. **A** HPASMCs were identified by immunofluorescence staining for α-SMA under normoxia. Scale bar = 50 μm. **B** Time-dependent changes in SOCS5 mRNA expression following HPASMCs under hypoxia (*n* = 3 each group). **C** Western blot analysis and quantification of SOCS5 protein expression in HPASMCs induced by hypoxia for 0, 6, 24 and 72 h. HPASMCs, human pulmonary arterial smooth muscle cells

Under normoxic conditions, HPASMCs were identified by IF staining for α-SMA (Fig. 2A). Treatment of HPASMCs with hypoxia upregulated SOCS5 mRNA expression in a time-dependent manner (3, 6 h) and downregulated in a time-dependent manner (12, 24, 48 and 72 h) (Fig. 2B). Western blot analysis further confirmed that the SOCS5 protein expression increased first and then decreased (Fig. 2C). Therefore, subsequent cell experiments were performed after normoxia or hypoxia treatment for 72 h.

### Overexpression of SOCS5 inhibited the abnormal proliferation and migration of HPASMCs under hypoxia in vitro, while knockdown of SOCS5 promoted it

**Fig. 3:**
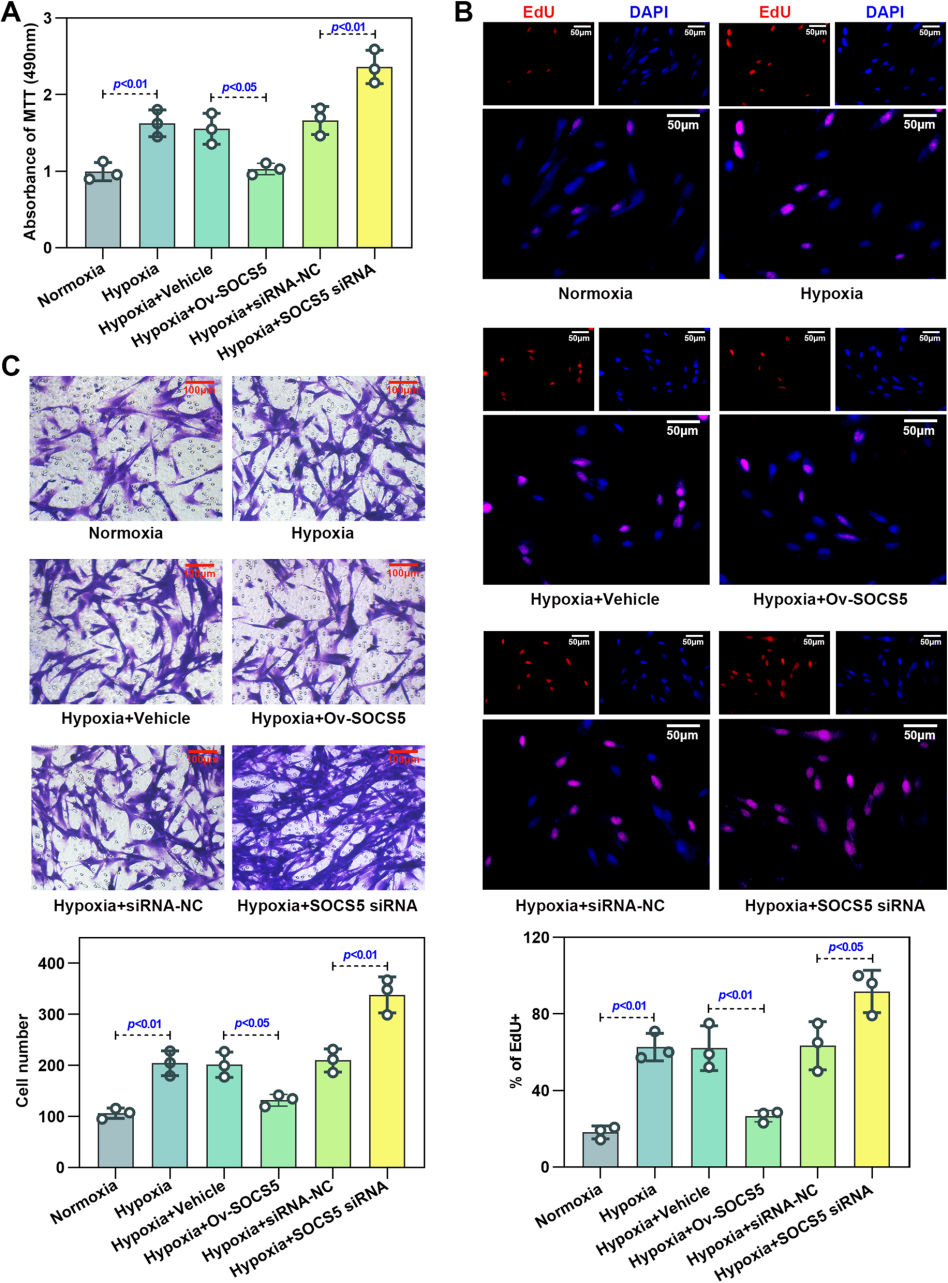
Effects of SOCS5 expression on HPASMC proliferation and migration under hypoxia conditions. **A**-**C** After HPASMCs were transfected with plasmids overexpressing or knocking down SOCS5 for 24 h, starved for 3 h and then treated with normoxia or hypoxia for 72 h, MTT was used to verify cell viability (**A**) (*n* = 3 each group). Proliferation of HPASMCs was evaluated by EdU incorporation assay and EdU-positive cells were quantified (**B**) (*n* = 3 each group). Scale bar = 50 μm. Migration of HPASMCs was assayed by transwell assay (**C**) (*n* = 3 each group). Scale bar = 100 μm. HPASMCs, human pulmonary arterial smooth muscle cells; RT-qPCR, quantitative reverse transcription polymerase chain reaction; MTT, 3- (4,5)-dimethylthiahiazo (-z-y1)-2,5-di- phenytetrazoliumromide; EdU, 5-Ethynyl-2’- deoxyuridine

To verify the efficiency of overexpression and knockdown of SOCS5, RT-qPCR and western blot was performed to measure the mRNA expression and protein level of SOCS5 in HPASMCs (Fig. S1A-D). The siRNA-3 with the best transfection efficiency was selected for the following experiments. As illustrated in Fig. 3, cell viability (Fig. 3A) and migration (Fig. 3C) were significantly elevated in hypoxia‐induced HPASMCs transfected with SOCS5 siRNA, whereas they were partly eliminated by transfection of Ov‐SOCS5. In addition, the percentage of EdU‐positive cells was found to be increased under hypoxia with SOCS5 knocking down and was reduced by SOCS5 overexpression (Fig. 3B).

### Overexpression of SOCS5 suppressed contraction of HPASMCs under hypoxia in vitro, while knockdown of SOCS5 accelerated it

**Fig. 4:**
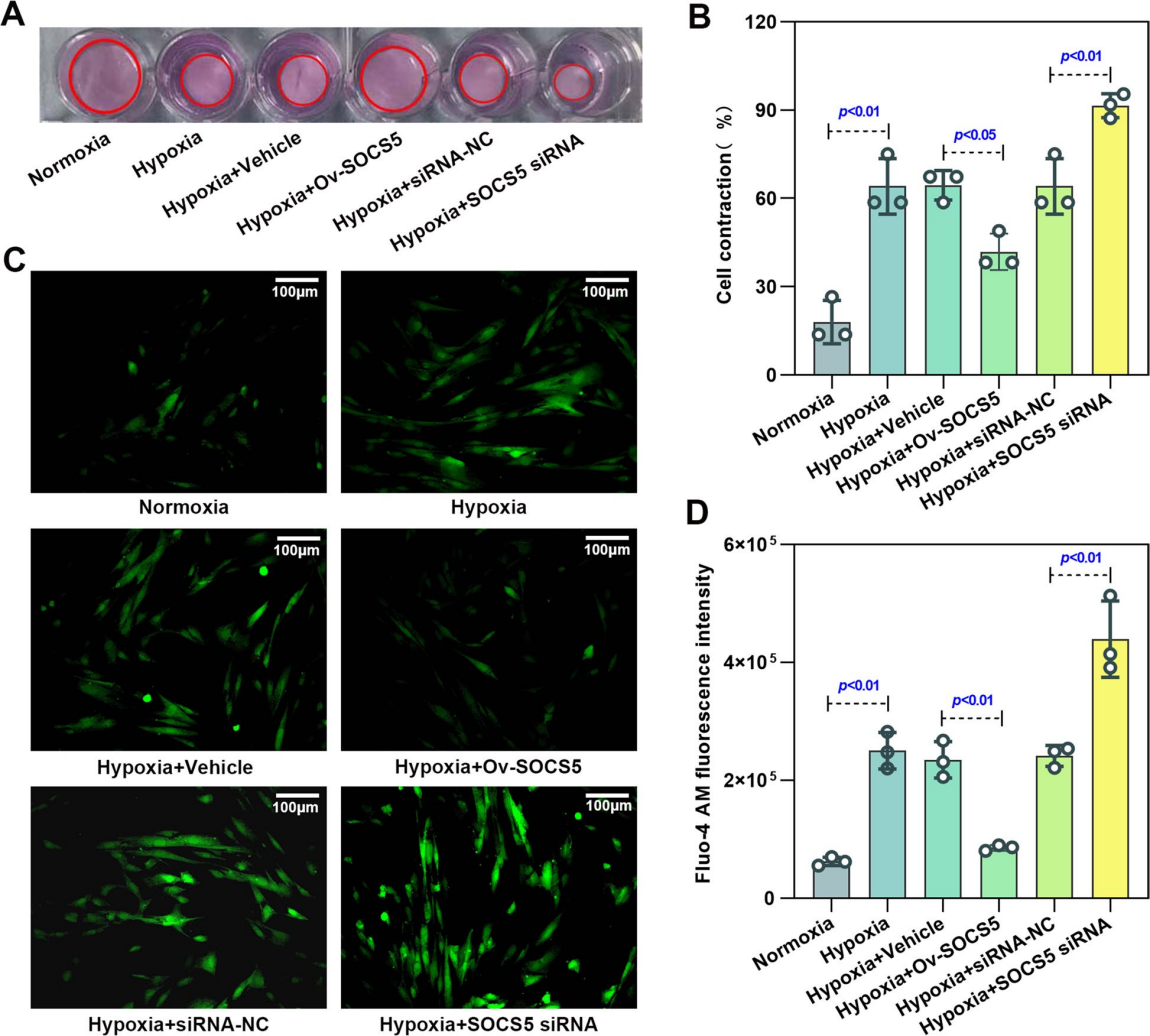
Effects of SOCS5 expression on HPASMC contractility under hypoxia conditions. **A** Representative image of cell contraction assay. The red circles represent the gel area. **B** Quantification of collagen contraction relative to the initial collagen gel area (*n* = 3 each group). **C** Representative images of [Ca^2+^]. Scale bar = 100 μm. **D** Quantitative analysis of Fluo-4 AM fluorescence intensity (*n* = 3 each group). Fluo-4 AM, a fluorescent dye. HPASMCs, human pulmonary arterial smooth muscle cells

Abnormal HPASMCs contraction under hypoxia is also prominent pathophysiological processes of PH. As shown in Fig. 4A and ​B, the contractile capability of hypoxia‐induced HPASMCs transfected with Ov-SOCS5, as assessed by the percentage of collagen gel size changes relative to the initial collagen gel size, was decreased compared to controls, whereas they were increased by transfection of SOCS5 siRNA. Overexpression of SOCS5 effectively restrained the abnormal contraction of HPASMCs under hypoxia. It has been shown that hypoxia disrupts the intracellular Ca^2+^ signaling dynamics, which leads to HPASMC dysfunction [25]. Elevated Ca^2+^ concentration promotes HPASMC contraction [26]. Subsequently, we evaluated Ca^2+^ signals by Fluo-4 AM (Fig. 4C&D), which further demonstrated our idea that high expression of SOCS5 inhibited HPASMC contraction.

### SOCS5 negatively regulated the JAK2/STAT3 signaling pathway in HPASMCs under hypoxia

**Fig. 5:**
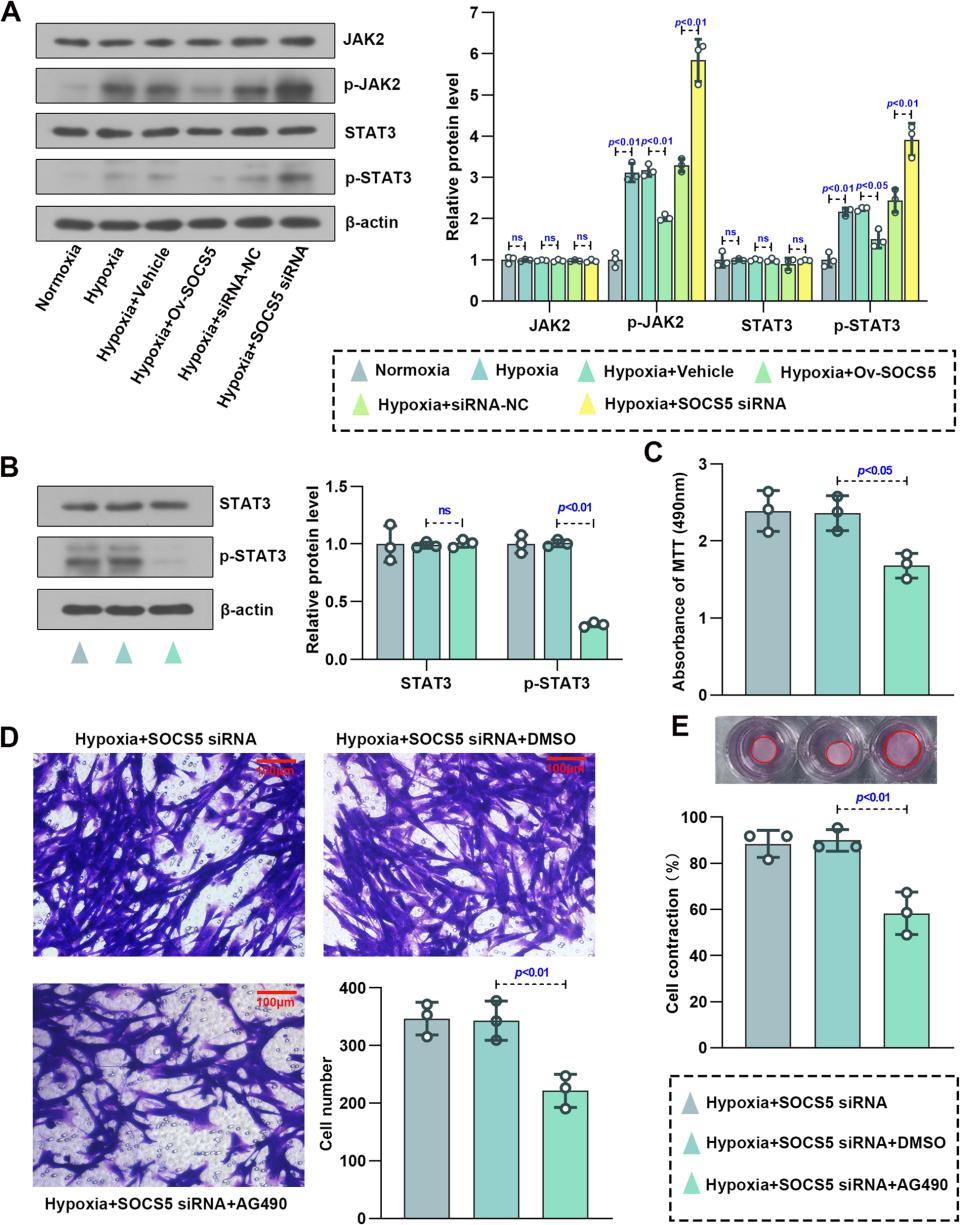
SOCS5 knockdown activated the JAK2/STAT3 signaling in HPASMCs. **A** Western blot analysis and quantification of JAK2, p-JAK2^Tyr1007/1008^, STAT3 and p-STAT3^Tyr705^ protein expression in HPASMCs, which were transfected with plasmids overexpressing or knocking down SOCS5 for 24 h, starved for 3 h and then treated with normoxia or hypoxia for 72 h. **B**-**E** After HPASMCs were transfected with plasmids knocking down SOCS5 for 24 h, starved for 3 h, supplemented with AG490 (10 μM) and then treated with hypoxia for 72 h, western blot analysis and quantification of STAT3 and p-STAT3^Tyr705^ protein expression were performed (**B**) (*n* = 3 each group). MTT was used to verify cell viability (**C**) (*n* = 3 each group). Migration of HPASMCs was assayed by transwell assay (**D**) (*n* = 3 each group). Representative image of cell contraction assay and quantification of collagen contraction relative to the initial collagen gel area was performed (**E**) (*n* = 3 each group). HPASMCs, human pulmonary arterial smooth muscle cells; MTT, 3- (4,5)-dimethylthiahiazo (-z-y1)-2,5-di- phenytetrazoliumromide

**Fig. 6:**
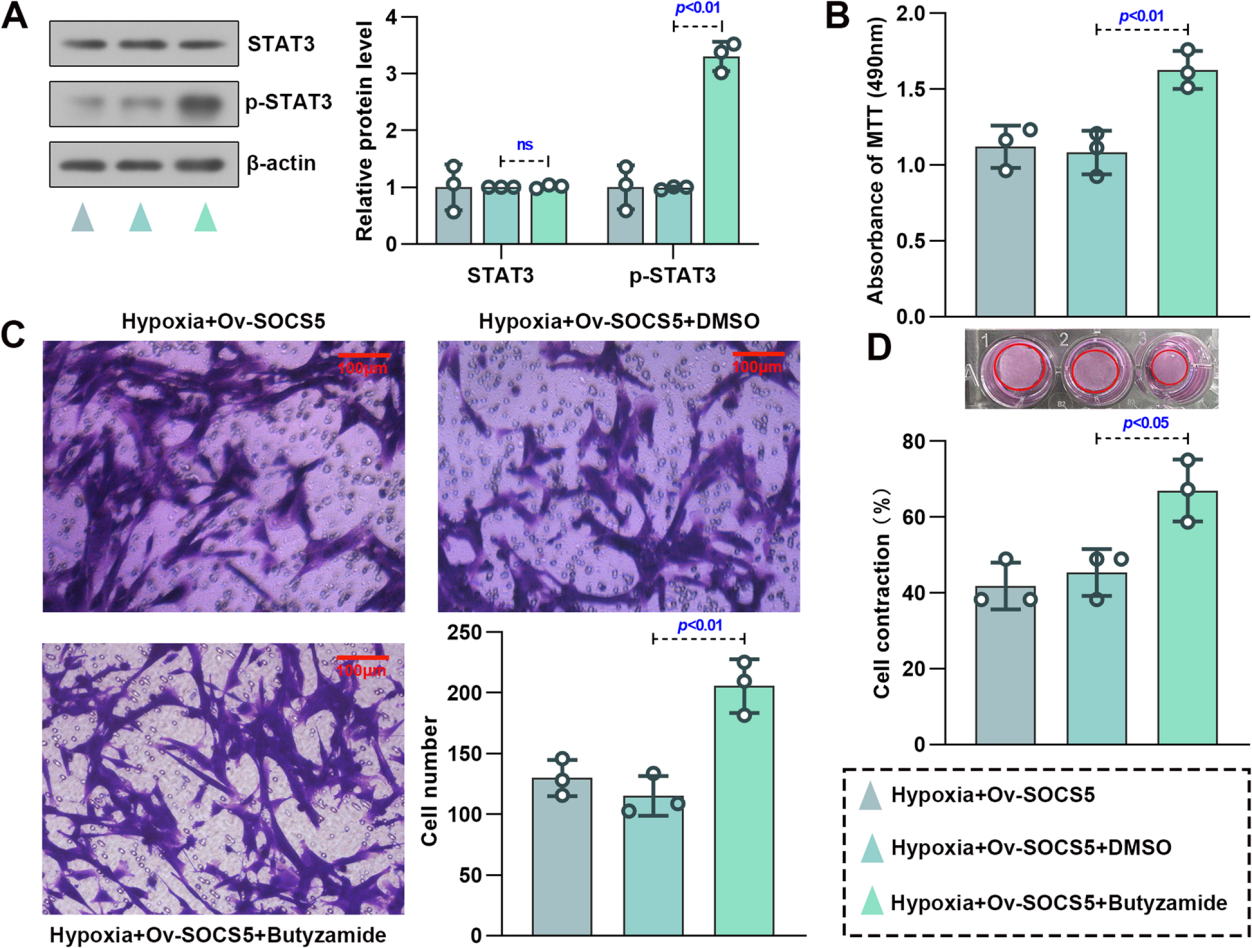
SOCS5 overexpression inhibited the JAK2/STAT3 signaling in HPASMCs. (A-D) After HPASMCs were transfected with plasmids overexpressing SOCS5 for 24 h, starved for 3 h, supplemented with Butyzamide and then treated with hypoxia for 72 h, western blot analysis and quantification of STAT3 and p-STAT3^Tyr705^ protein expression were performed (**A)** (*n* = 3 each group). MTT was used to verify cell viability (**B**) (*n* = 3 each group). Migration of HPASMCs was assayed by transwell assay (**C**) (*n* = 3 each group). Representative image of cell contraction assay and quantification of collagen contraction relative to the initial collagen gel area was performed (**D**) (*n* = 3 each group). HPASMCs, human pulmonary arterial smooth muscle cells; MTT, 3- (4,5)-dimethylthiahiazo (-z-y1)-2,5-di- phenytetrazoliumromide

JAK2/STAT3 pathway is one of the signaling pathways involved in the development of PH [15]. To determine the activity of JAK2/STAT3 signaling, the phosphorylation of JAK2 and STAT3 was assessed (Fig. 5A). Overexpression of SOCS5 inhibited activation of the JAK2/STAT3 signaling pathway in HPASMCs under hypoxia, while knockdown of SOCS5 partially reversed it. AG490, a JAK2 inhibitor, and Butyzamide, a JAK2 activator, were used to the validation in hypoxia‐induced HPASMCs transfected with SOCS5 siRNA and overexpressing SOCS5, respectively. Results showed that the phosphorylation of STAT3 was restrained in hypoxia‐induced HPASMCs transfected with SOCS5 siRNA and accompanied by addition of AG490 (Fig. 5B). Moreover, abnormal proliferation, migration and contraction of hypoxia‐induced HPASMCs were also significantly suppressed (Fig. 5C-E). However, opposite results were obtained from hypoxia‐induced HPASMCs transfected with overexpressing SOCS5 and accompanied by addition of Butyzamide (Fig. 6A-D). These results suggested that SOCS5 inhibited JAK2 activation, subsequently leading to a decrease in STAT3 phosphorylation. Furthermore, SOCS5 affected hypoxia‐induced HPASMCs proliferation, migration and contraction by negatively regulating JAK2/STAT3 pathway.

### SOCS5 was negatively regulated by miR-155-5p, which in turn promoted abnormal proliferation, migration and contraction of HPASMCs under hypoxia

**Fig. 7:**
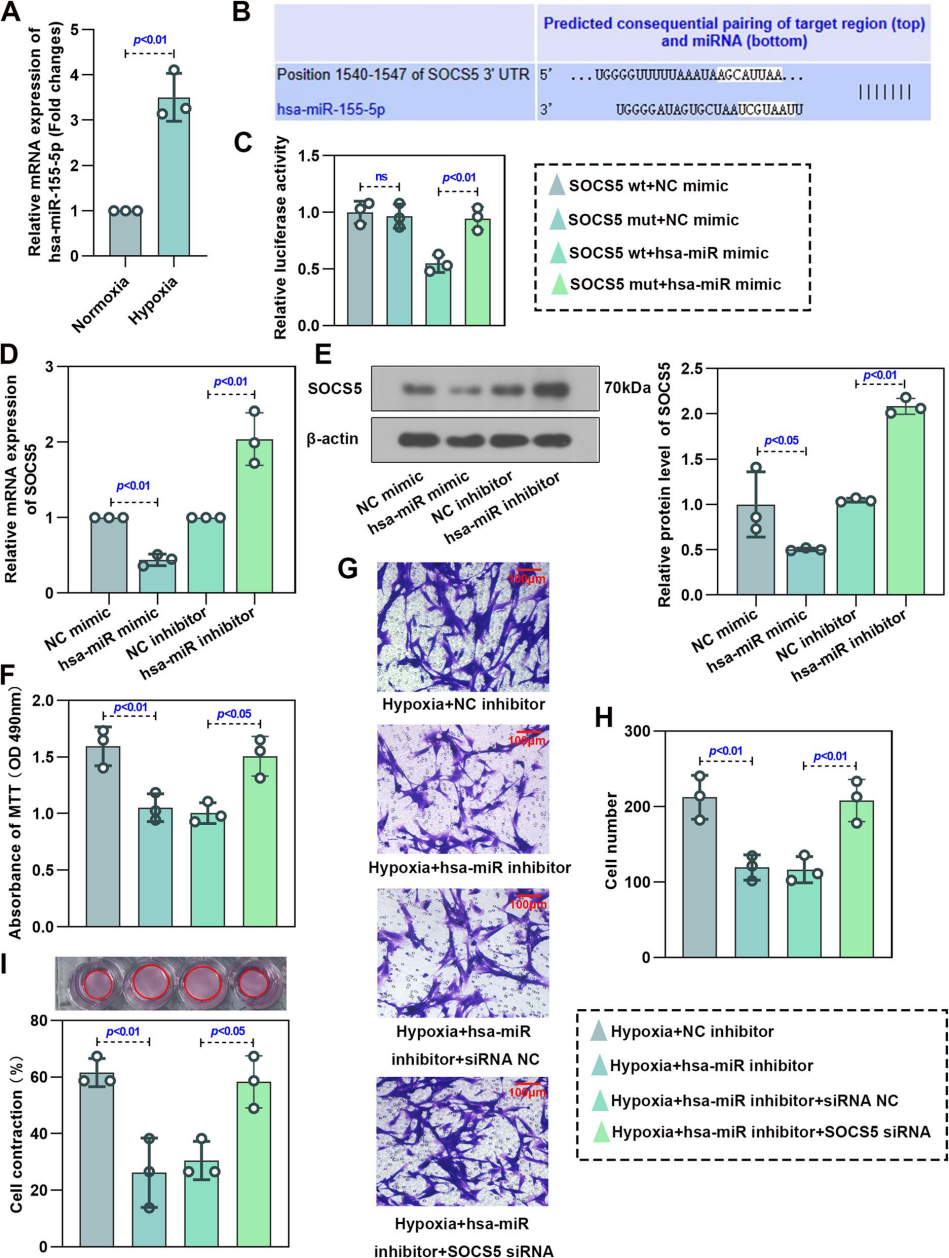
SOCS5 was regulated by hsa-miR-155-5p. **A** RT-qPCR was performed to detect hsa-miR-155-5p mRNA expression in HPASMCs cultured under normoxia or hypoxia conditions (*n* = 3 each group). **B** Prediction of the relationship between hsa-miR-155-5p and SOCS5. **C** Luciferase activity of 293 T cells transfected with plasmids carrying a wild-type or mutant 3′UTR of SOCS5 in response to hsa-miR-155-5p mimic. **D**-**E** RT-qPCR and western blot analysis of the expression of SOCS5 in HPASMCs transfected with plasmids carrying hsa-miR-155-5p mimic or inhibitor (*n* = 3 each group). **F**-**I** After HPASMCs were transfected with plasmids carrying hsa-miR-155-5p inhibitor and knocking down SOCS5 for 24 h, starved for 3 h and then treated with hypoxia for 72 h, MTT was used to verify cell viability (**F**) (*n* = 3 each group). Migration of HPASMCs was assayed by transwell assay (**G**&**H**) (*n* = 3 each group). Representative image of cell contraction assay and quantification of collagen contraction relative to the initial collagen gel area was performed (**I**) (*n* = 3 each group). RT-qPCR, quantitative reverse transcription polymerase chain reaction; HPASMCs, human pulmonary arterial smooth muscle cells; MTT, 3- (4,5)-dimethylthiahiazo (-z-y1)-2,5-di- phenytetrazoliumromide

The expression of miR-155-5p was observed to be markedly increased in hypoxia‐induced HPASMCs compared with cells under normoxia (Fig. 7A). In addition, through the database (miRDB & TargetScan website), SOCS5 was found to be a target of miR-155-5p (Fig. 7B). We then explored the relationship between miR-155-5p and SOCS5. The results of dual-luciferase report assay illustrated that luciferase activity of SOCS5 wt + miR-155-5p mimic group was significantly reduced as compared with SOCS5 mut + miR-155-5p mimic group, but did not change obviously in SOCS5 wt/mut + miR-155-5p mimic group (Fig. 7C). RT-qPCR and western blot analyses were further demonstrated SOCS5 was negatively regulated by miR-155-5p (Fig. 7D-E). The proliferation, migration and contraction of hypoxia‐induced HPASMCs was significantly suppressed by miR-155-5p inhibitor treatment, but was greatly enhanced by transfection of SOCS5 siRNA (Fig. 7F-H). These data suggested that miR-155-5p directly targeted SOCS5 in HPASMCs and regulated abnormal proliferation, migration and contraction of HPASMCs.

## Discussions

PH is a complex progressive disease, and it is imperative to understand its molecular pathogenesis. In this study, we investigated the potential role of SOCS5 in experimental pulmonary hypertension and underlying mechanisms. Our results indicated that SOCS5 expression was lower in PH mice than normal ones and SOCS5 inhibited abnormal proliferation, migration and contraction of HPASMCs under hypoxia in vitro. Mechanistically, we revealed that miR-155-5p directly targeted SOCS5 to activate JAK2/STAT3 signaling pathway.

The SOCS family is involved in the negative feedback regulation of cytokine signaling transduction. SOCS proteins have been linked to a range of inflammatory diseases, autoimmune diseases, and immune-related cancers [27]. During chronic obstructive pulmonary disease progression, miR-132 overexpression targeted and inhibited SOCS5, thereby promoting EGFR protein expression and inflammatory cytokine production in human mononuclear cells [14]. Upregulation of SOCS5 was associated with inhibition of cell viability, migration and invasion in non‐small cell lung cancer [28]. In our study, SOCS5 expression was significantly decreased in lung tissue of PH mice. Consistent with our results, SOCS5 was significantly reduced in the lung tissues of severe acute pancreatitis-induced acute lung injury rats [29]. This suggests that SOCS5 may also play a negative regulatory role in PH.

Pulmonary vascular remodeling mainly occurs in pulmonary artery smooth muscle cells (PASMCs), endothelial cells and fibroblasts in the vascular wall. Excessive PASMCs proliferation and migration tends to cause pulmonary vascular remodeling and trigger PH [30]. In this study, we found that SOCS5 expression was low in PASMCs, and overexpression of SOCS5 inhibited the abnormal proliferation and migration of HPASMCs under hypoxia in vitro, while knockdown of SOCS5 promoted it. In addition, SOCS5 expression was linked to the regulation of pulmonary vascular tone.

**Fig. 8:**
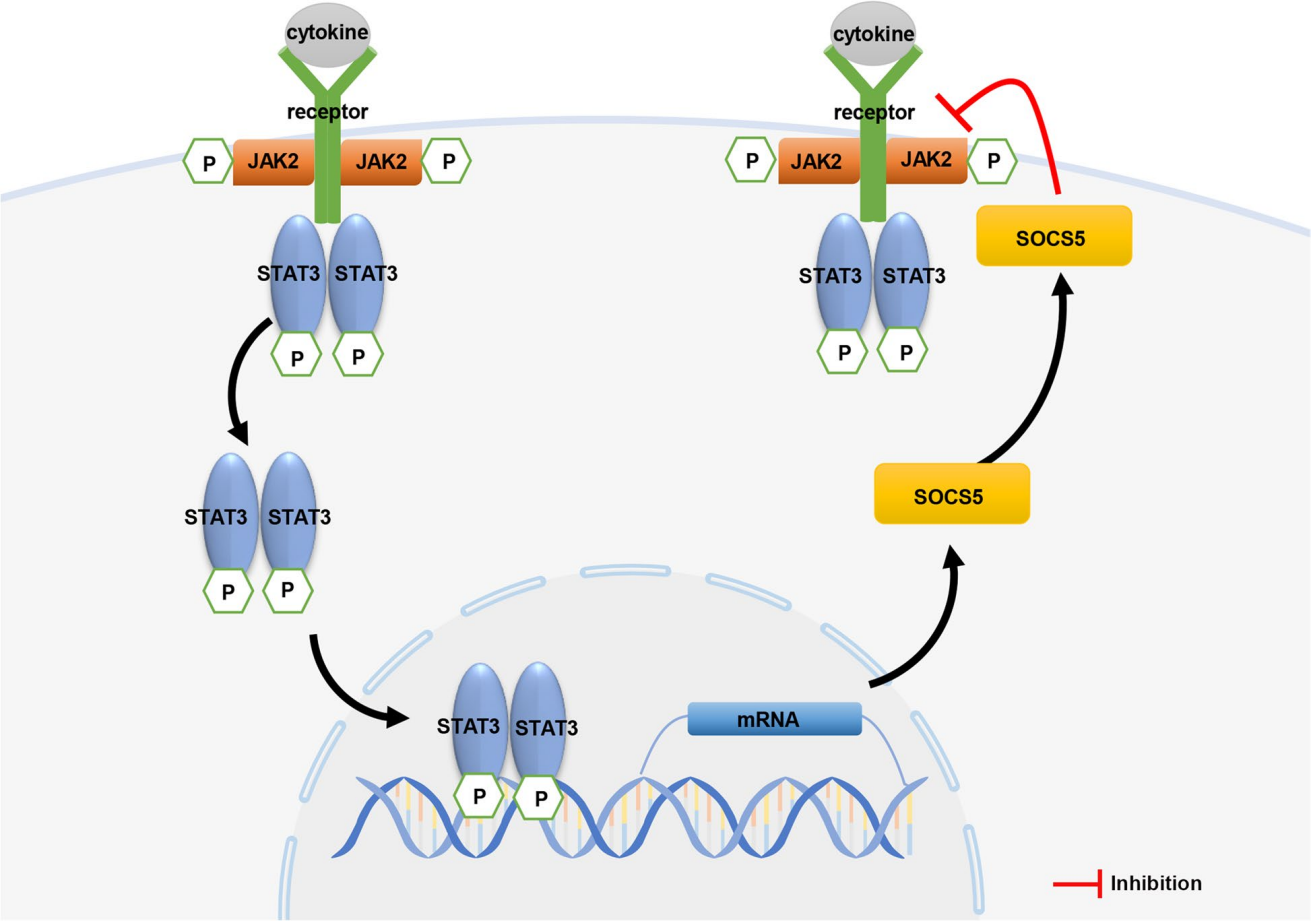
Mechanism of SOCS5 inhibition of JAK2/STAT3 signaling pathway

SOCS5 is involved in the cytokine signaling process through the JAK2/STAT3 pathway [8] (Fig. 8). After cytokines bind to their corresponding receptors, JAK kinases are recruited to the receptors and activated. Activated JAK catalyzes tyrosine phosphorylation of the receptor. Through the recognition and binding of SH2 domain of phosphorylated STAT molecule, STAT forms a dimer and enters the nucleus. As an active transcription factor, STAT dimer directly affects the expression of SOCS5, which in turn affects cell proliferation or differentiation [31]. A previous study showed that Leonurine plays an anti-leukemia role by inhibiting the proliferation, migration and colony formation of chronic myeloid leukemia cells and promoting their apoptosis through the miR-18a-5p/SOCS5/JAK2/STAT3 axis [11]. Similarly, we demonstrated that SOCS5 negatively regulated the JAK2/STAT3 signaling pathway in HPASMCs under hypoxia. Exploring the SOCS5-mediated JAK2/STAT3 pathway will help us better understand the mechanism of SOCS5 in PH.

miRNAs play a key role in gene expression, mediating the occurrence and development of many diseases, including a variety of tumors [32], hypertension [33] and PH [34]. miRNAs induce the proliferation and migration of smooth muscle cells (SMCs) by regulating the expression levels of related genes, and participate in PH occurrence [35]. miR-17–92, miR-21, miR-145, miR-210 are all considerably upregulated and miR-124, miR-210 are downregulated in the context of PH [36]. Recent studies have shown that miR-155-5p regulated hypoxia-induced PASMC function by targeting PYGL [37]. In our study, the expression of miR-155-5p was observed to be markedly increased in hypoxia‐induced HPASMCs compared with cells under normoxia. Bioinformatics website suggested that SOCS5 might be a downstream target of miR-155-5p, which was confirmed by dual luciferase activity experiments. In vitro, miR-155-5p overexpression suppressed SOCS5 expression, and miR-155-5p inhibition instead promoted SOCS5 expression. In severe acute pancreatitis induced acute lung injury, stellate ganglion block promoted the expression of SOCS5, inhibited the expression of miR-155-5p, and inhibited the activation of JAK2/STAT3 pathway [29]. Our study also demonstrated that SOCS5 was negatively regulated by miR-155-5p, which in turn promoted abnormal proliferation, migration and contraction of HPASMCs under hypoxia.

In this study, SOCS1 was also downregulated in the mouse lung tissue on hypoxia exposure compared with normoxia controls. In addition, SOCS1 also have inhibitory effect on JAK2-STAT3 signaling pathway activation in PH [38] and has also been reported to be a target of miR-155-5p [39, 40]. Maybe, SOCS1 and SOCS5 have redundant roles in the miR155-5p/SOCS/JAK2/STAT3 pathway in PH. This requires further verification.

To summarize, we have uncovered that the decrease of SOCS5 relieved the inhibitory effect on JAK2/STAT3 pathway and promoted the development of PH through promoting the abnormal proliferation, migration and contraction of HPASMCs. Moreover, SOCS5 was negatively regulated by miR-155-5p. These findings may provide new ideas for the treatment strategy of PH.

There is a limitation to our study. The role of SOCS5 in PH and its mechanism were primarily investigated at the cellular level. Therefore, further investigations in the animal model of PH are expected to provide more reliable evidence for our findings.

### Supplementary Information

**Additional file 1:** **Figure S1. **(A-D) RT-qPCR and western blot verification of the expression of overexpressing (A&B) or knocking down (C&D) SOCS5 in HPASMCs (results were normalized to control) (*n* = 3 each group).

**Additional file 2:** **Figure S2. **(A) Representative photographs of immunofluorescence staining for SOCS5 and CD31 in whole lungs of control and PH mice. (B) Representative photographs of immunofluorescence staining for SOCS5 and Vimentin in whole lungs of control and PH mice. Scale bar = 50 μm. PH, pulmonary hypertension.

**Additional file 3:** **Supplementary material.** The original images in triplicate of western blotting in this study . The blots were cropped prior to hybridization with primary antibodies.

## Data Availability

All the data supporting the findings of this study are available from the corresponding author upon reasonable request.
