# Supplementary figures and images for: SOCS5, targeted by miR-155-5p, plays a negative regulatory role in pulmonary hypertension through inhibiting JAK2/STAT3 signaling pathway
Source: BMC Pulm Med. 2024 Jan 24;24:52. doi: 10.1186/s12890-024-02857-6 (PMC10809471; doi:10.1186/s12890-024-02857-6)

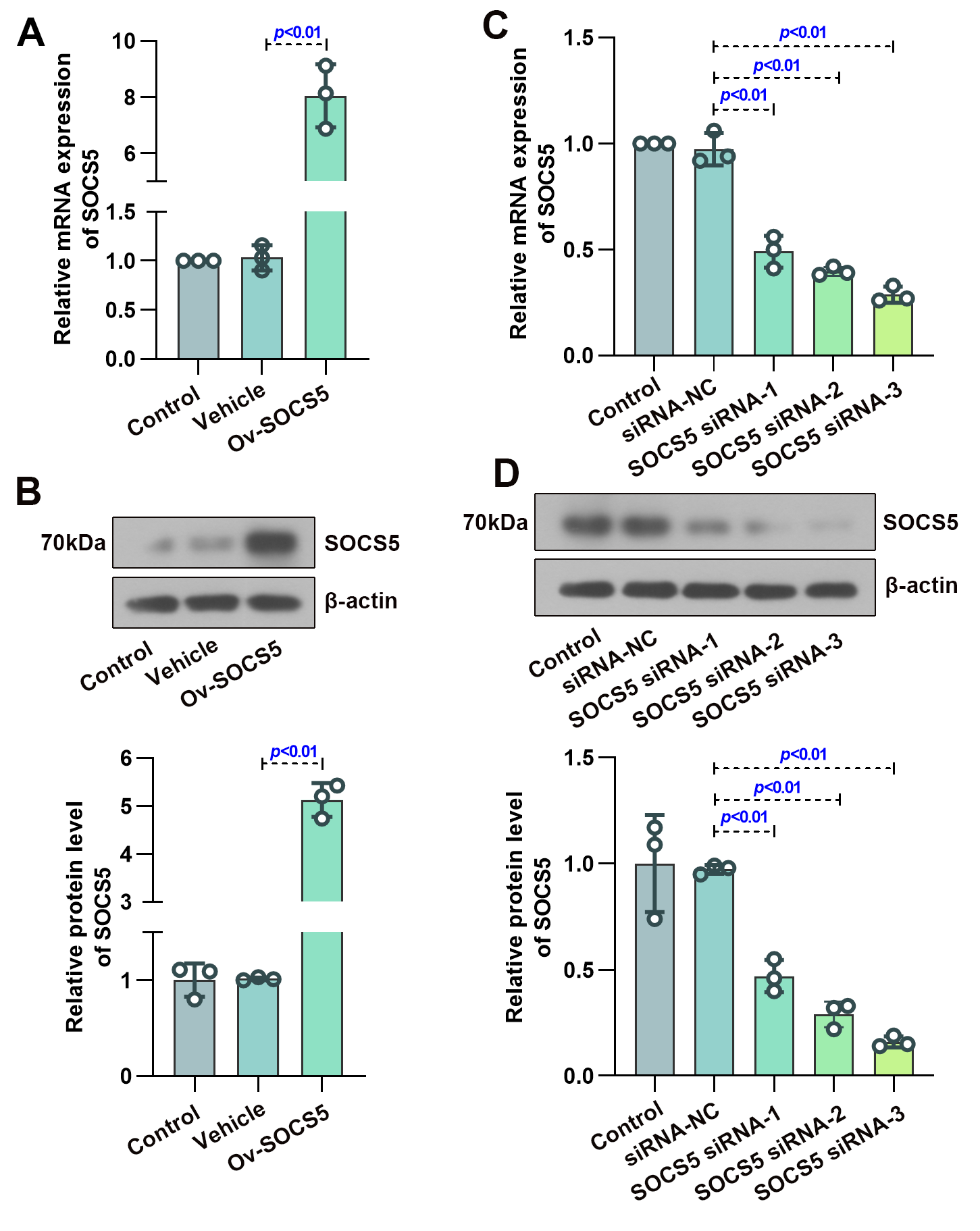

Supplement: Supplementary file 1 — Additional file 1: Figure S1. (A-D) RT-qPCR and western blot verification of the expression of overexpressing (A&B) or knocking down (C&D) SOCS5 in HPASMCs (results were normalized to control) (n = 3 each group). [file 12890_2024_2857_MOESM1_ESM.tif]

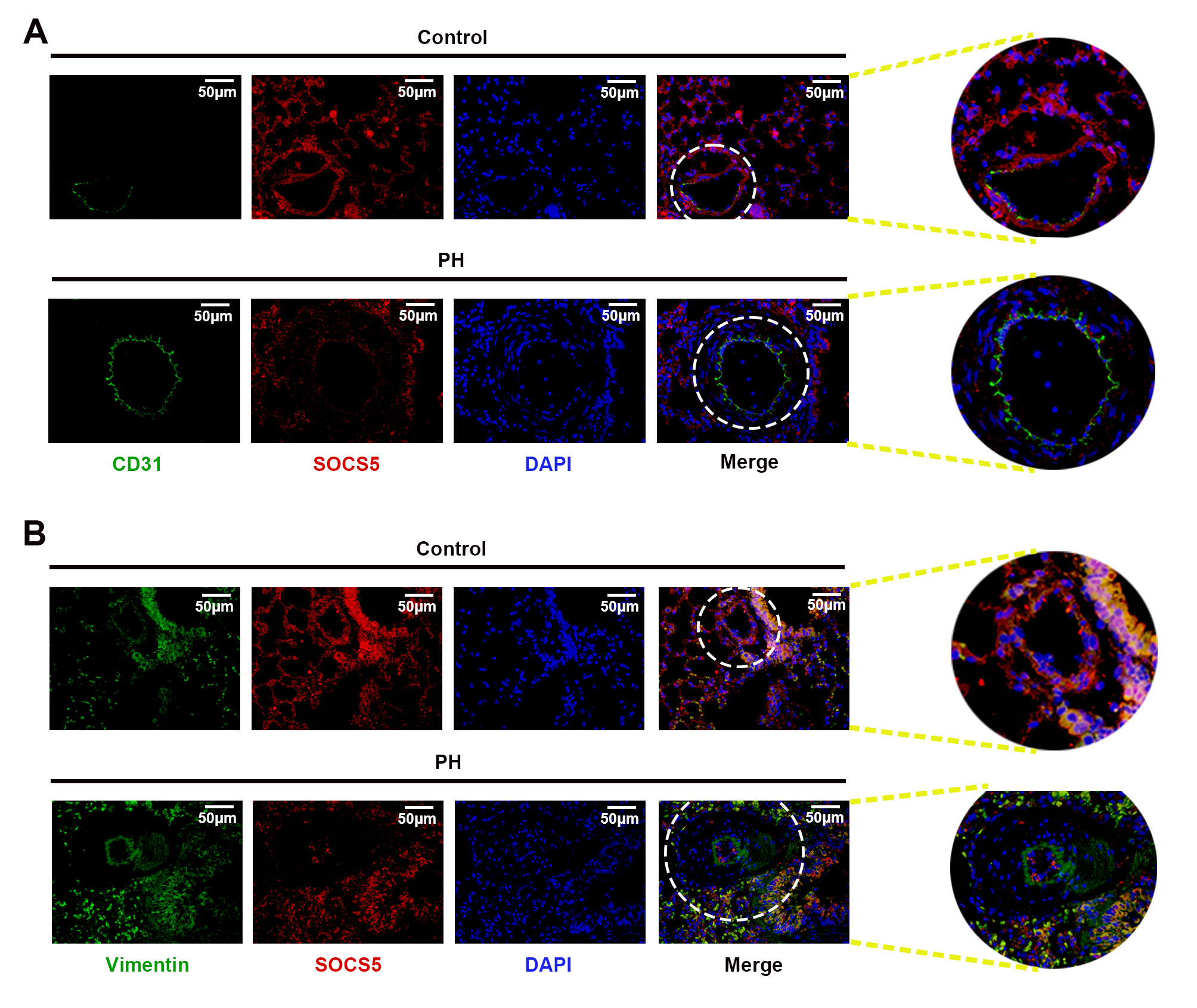

Supplement: Supplementary file 2 — Additional file 2: Figure S2. (A) Representative photographs of immunofluorescence staining for SOCS5 and CD31 in whole lungs of control and PH mice. (B) Representative photographs of immunofluorescence staining for SOCS5 and Vimentin in whole lungs of control and PH mice. Scale bar = 50 μm. PH, pulmonary hypertension. [file 12890_2024_2857_MOESM2_ESM.tif]
