# Supplementary material for: SOCS5, targeted by miR-155-5p, plays a negative regulatory role in pulmonary hypertension through inhibiting JAK2/STAT3 signaling pathway
Source: BMC Pulm Med. 2024 Jan 24;24:52. doi: 10.1186/s12890-024-02857-6 (PMC10809471; doi:10.1186/s12890-024-02857-6)
Supplement: Supplementary file 3 — Additional file 3: Supplementary material. The original images in triplicate of western blotting in this study . The blots were cropped prior to hybridization with primary antibodies. [file 12890_2024_2857_MOESM3_ESM.pdf]

Figure 1

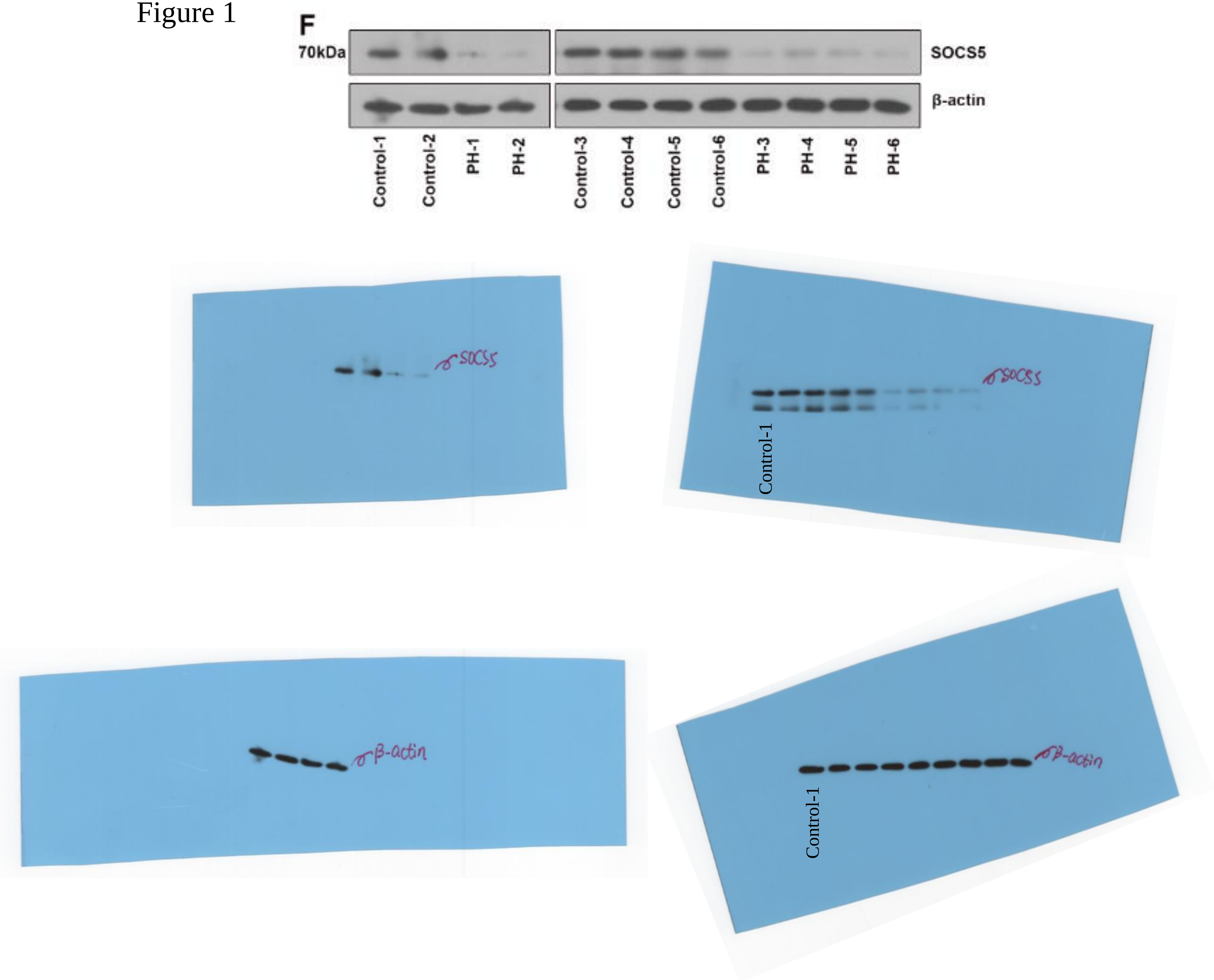
F
70kDa
SOCS5
β-actin
Control-1
Control-2
PH-1
PH-2
Control-3
Control-4
Control-5
Control-6
PH-3
PH-4
PH-5
PH-6
Control-1
Control-1

Figure 2

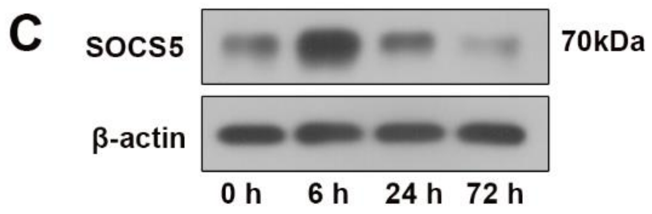
C
SOCS5
70kDa
β-actin
0 h
6 h
24 h
72 h

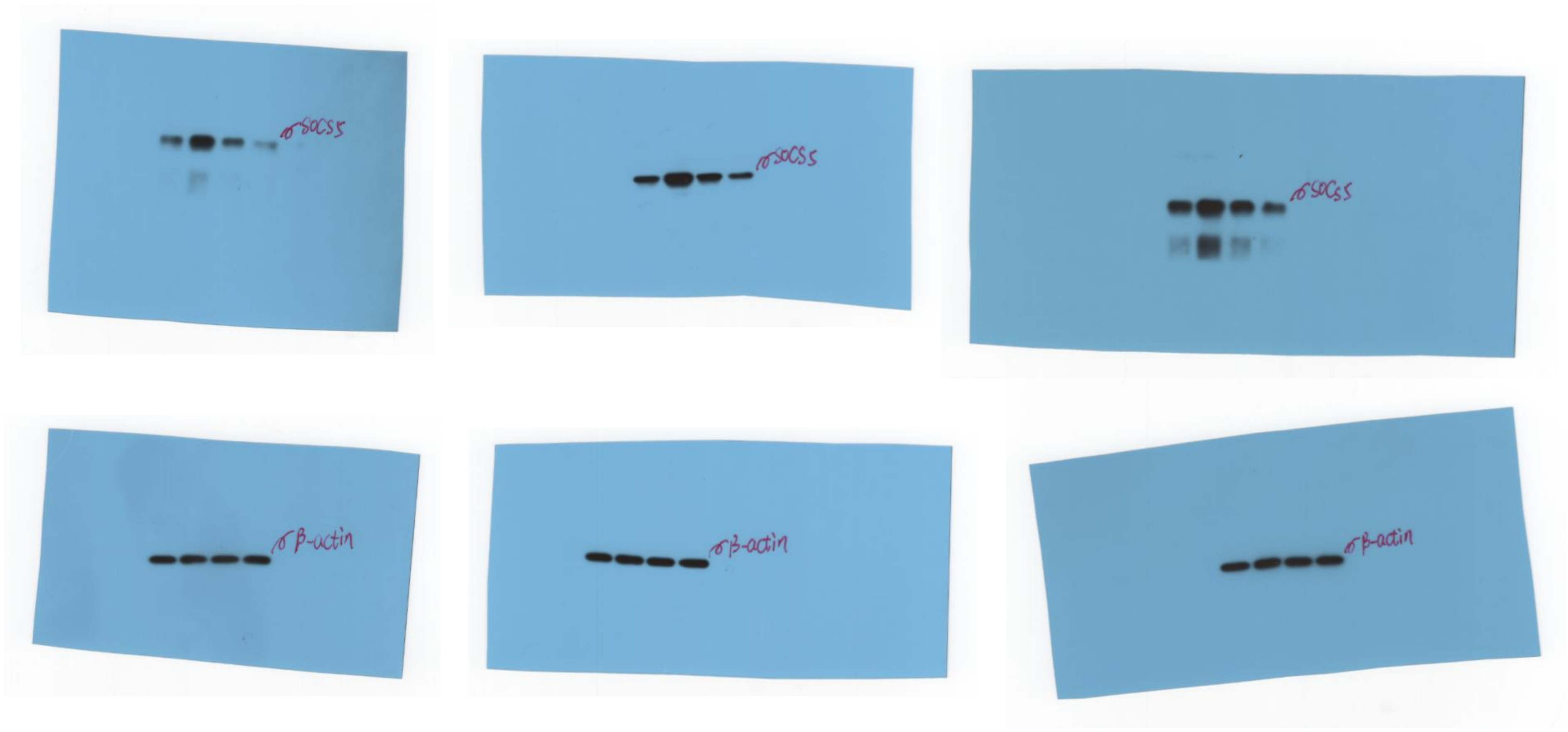
SOCS5
SOCS5
SOCS5
β-actin
β-actin
β-actin

Figure 5

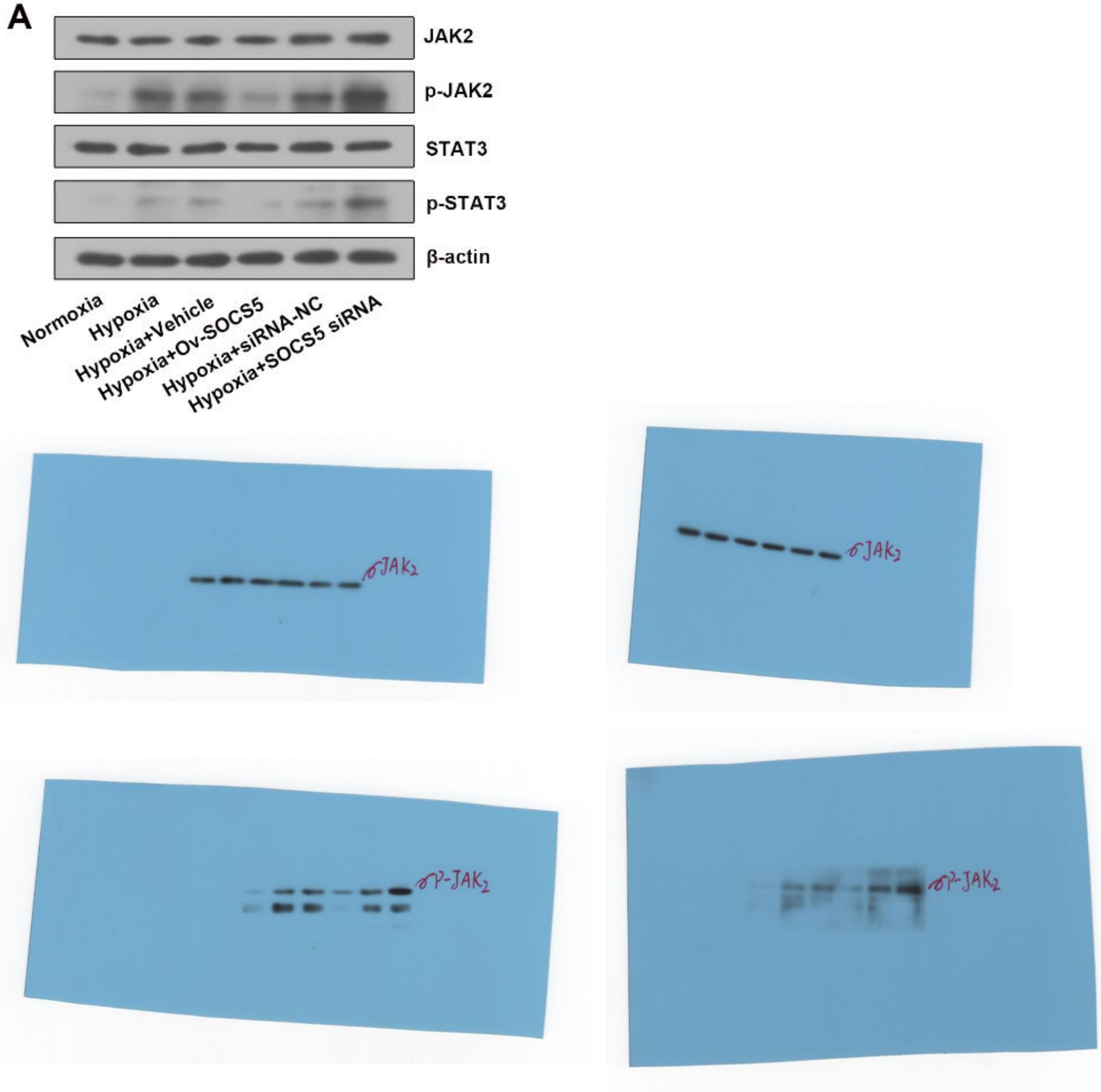
A
JAK2
p-JAK2
STAT3
p-STAT3
β-actin
Normoxia
Hypoxia
Hypoxia+Vehicle
Hypoxia+Ov-SOCS5
Hypoxia+siRNA-NC
Hypoxia+SOCS5 siRNA
JAK2
JAK2
JAK2
P-JAK2
P-JAK2
P-JAK2

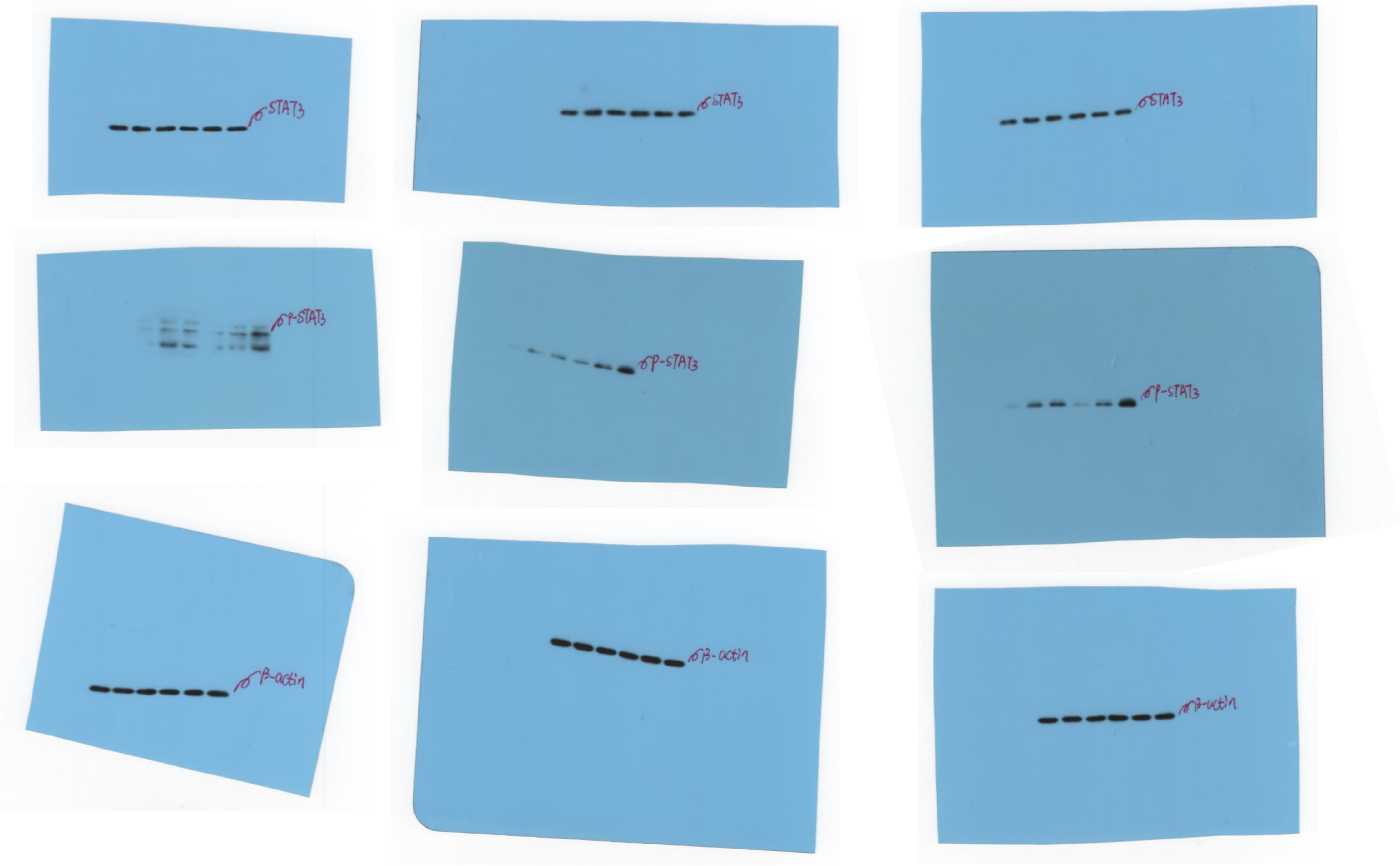

STAT3
STAT3
STAT3
p-STAT3
p-STAT3
p-STAT3
β-actin
β-actin
β-actin

Figure 5

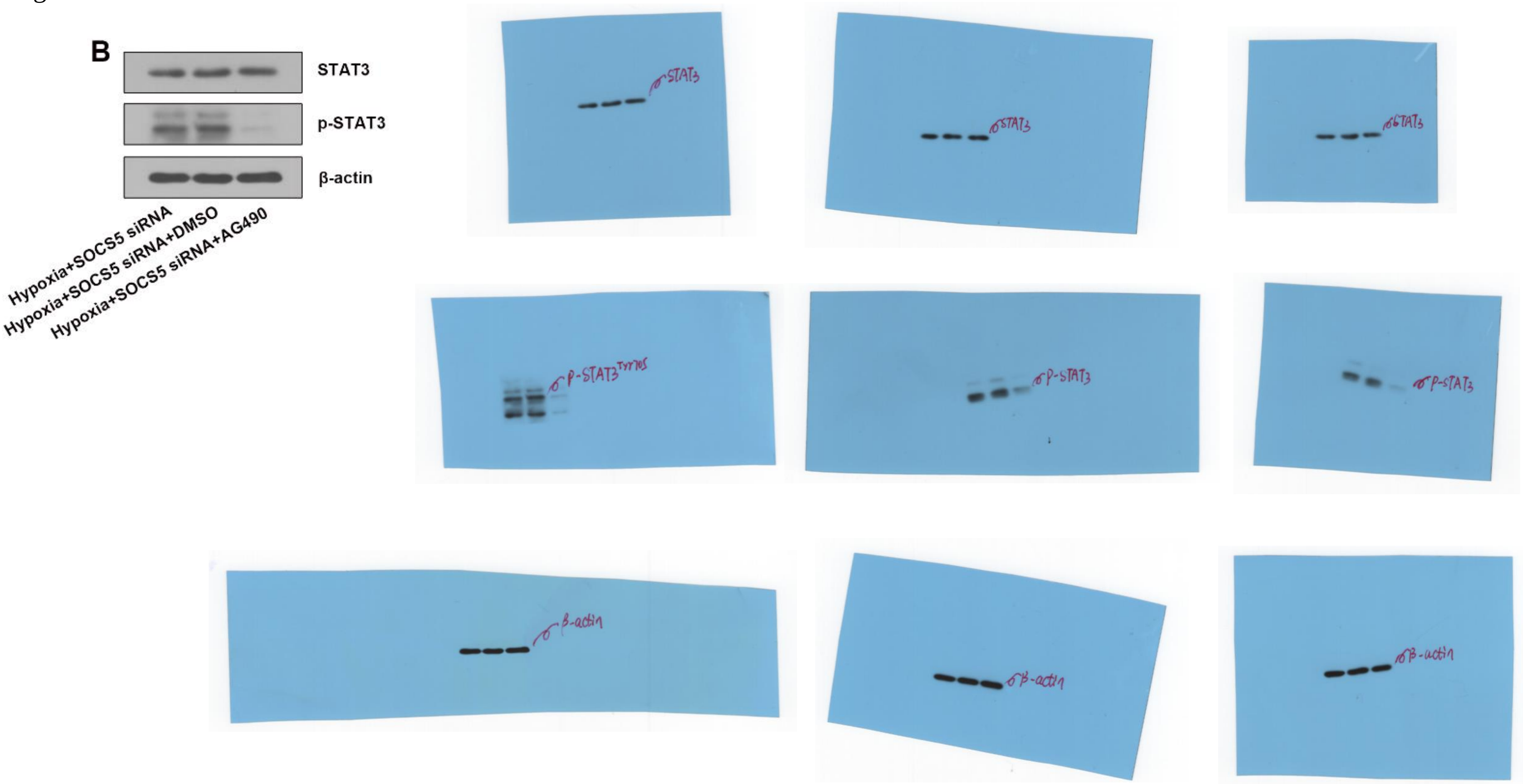
B
STAT3
p-STAT3
β-actin
Hypoxia+SOCS5 siRNA
Hypoxia+SOCS5 siRNA+DMSO
Hypoxia+SOCS5 siRNA+AG490
STAT3
STAT3
STAT3
p-STAT3 Tyr705
p-STAT3
p-STAT3
β-actin
β-actin
β-actin

Figure 6

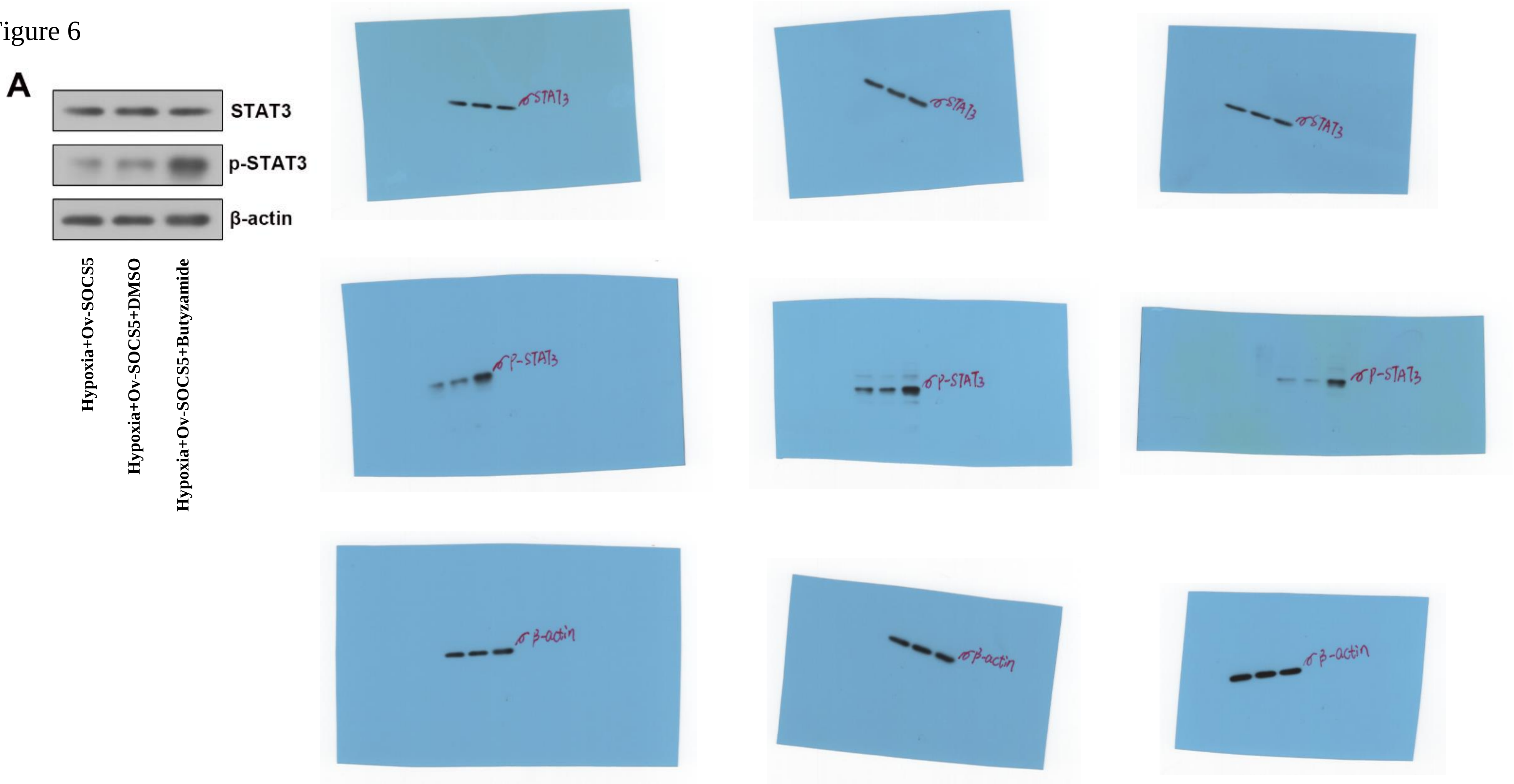

A
STAT3
p-STAT3
β-actin
Hypoxia+Ov-SOCS5
Hypoxia+Ov-SOCS5+DMSO
Hypoxia+Ov-SOCS5+Butyzamide

Figure 7

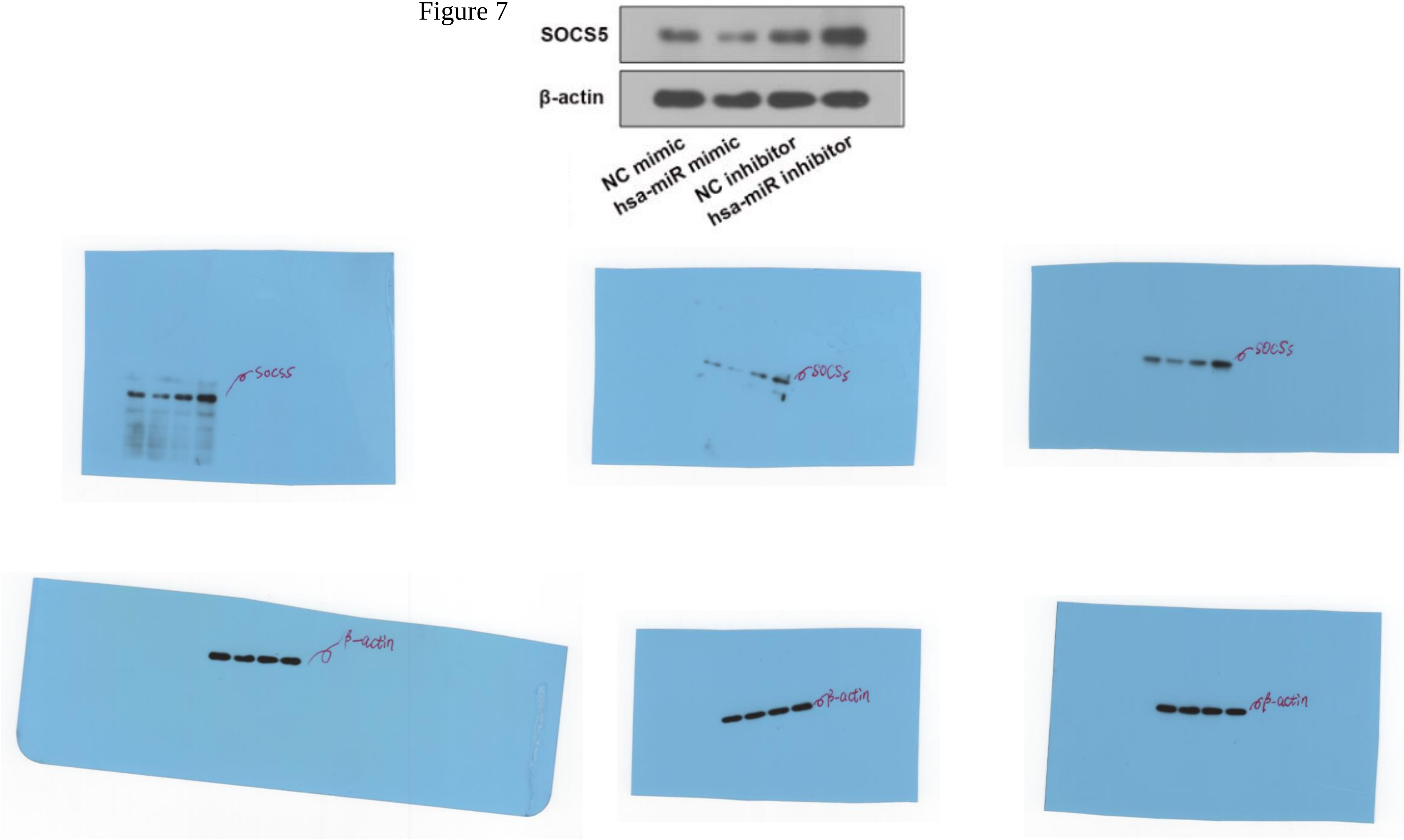
SOCS5
β-actin
NC mimic
hsa-miR mimic
NC inhibitor
hsa-miR inhibitor
SOCS5
SOCS5
SOCS5
β-actin
β-actin
β-actin

Figure S1

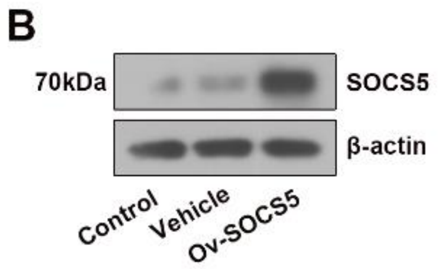
B
70kDa
SOCS5
β-actin
Control
Vehicle
Ov-SOCS5

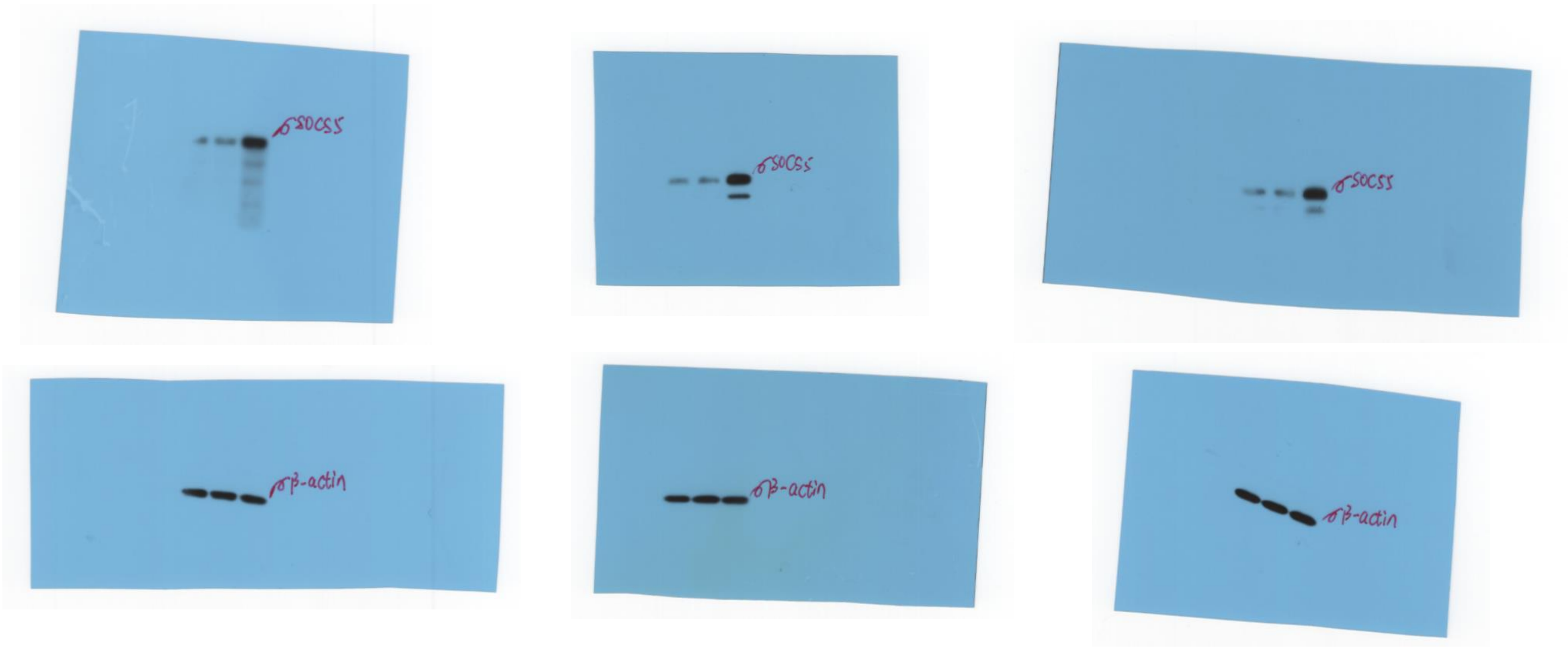
SOCS5
SOCS5
SOCS5
β-actin
β-actin
β-actin

Figure S1 **D**

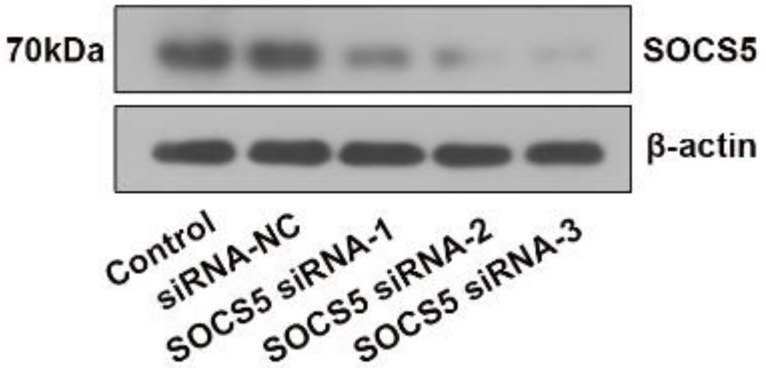

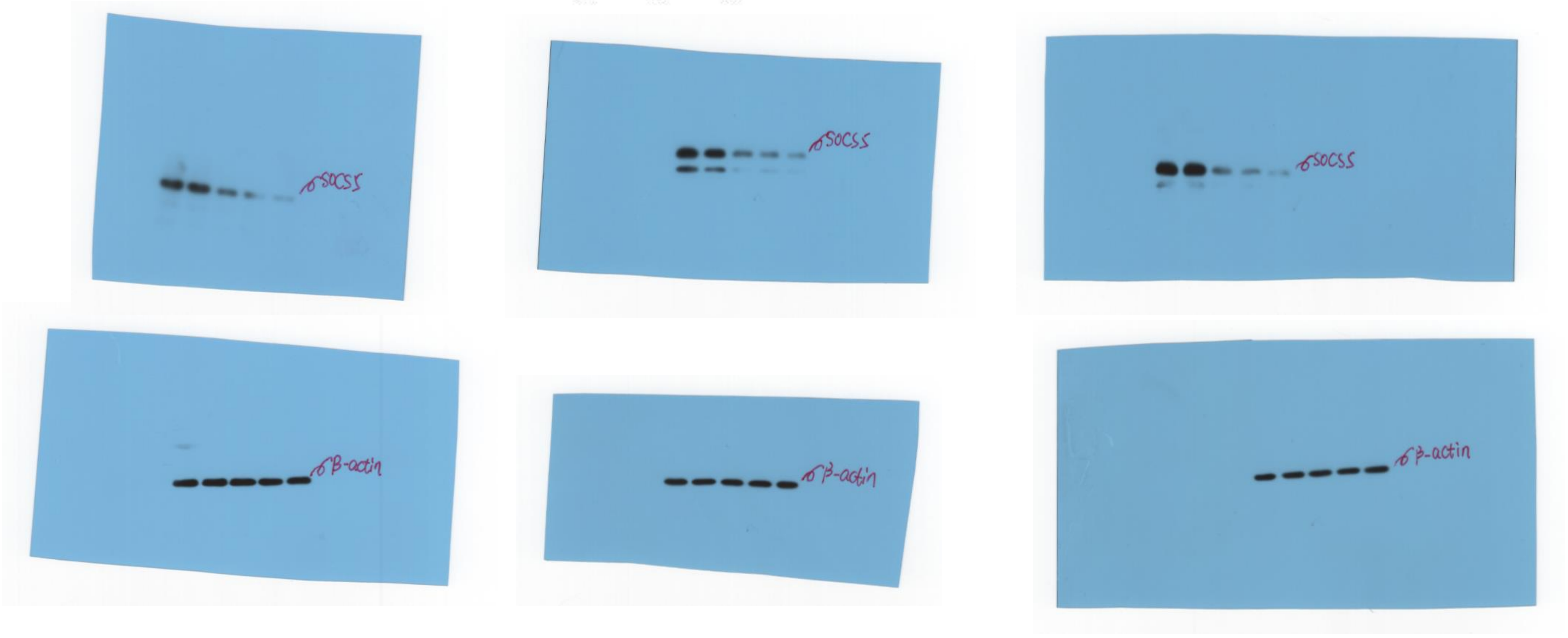

Supplementary material: The original images in triplicate of western blotting in this study . The blots were cropped prior to hybridization with primary antibodies.
